# Supplementary material for: Newly designed amplicons-based method for near-full-length genome (NFLG) sequencing of HIV-1 group M recombinant forms
Source: Mol Biol Rep. 2025 Apr 4;52(1):362. doi: 10.1007/s11033-025-10470-x (PMC11971223; doi:10.1007/s11033-025-10470-x)
Supplement: Supplementary file 1 — Supplementary file1 (DOCX 6784 KB) [file 11033_2025_10470_MOESM1_ESM.docx]

**Supplementary Material**

**Supplement table 1. Primers designed in this study to generate the amplicons for HIV-1 NFLG sequencing**

| **Primer Name** | **Pool** | **Sequence** | **Start**  (HXB2) | **END**  (HXB2) | **Size (bp)** | **% GC** | **TM (°C)** | **strand** | **PCR**  **product (bp)** |
| --- | --- | --- | --- | --- | --- | --- | --- | --- | --- |
| SC_CON_3_471_F | 1 | CCAGATCTGAGCCTGGGA | 471 | 487 | 18 | 61.1 | 57.6 | + | 319 |
| SC_CON_3_789_R | 1 | GACGCTCTCGCACCCATC | 789 | 806 | 18 | 67 | 60.6 | - |  |
| SC_CON_5_997_F | 1 | ACAGGAWCAGARGARMTTARATCATTAT | 997 | 1024 | 28 | 29.2 | 55.9-61.5 | + | 359 |
| SC_CON_5_1369_R | 1 | CATRGCTGCYTGATGTCCCC | 1364 | 1383 | 20 | 61.1 | 58.4-63.3 | - |  |
| SC_CON_7_1630_F | 1 | AGCATTYTGGAYATAARACAAGGRC | 1630 | 1654 | 25 | 38.1 | 56.4-63.1 | + | 385 |
| SC_CON_7_2019_R | 1 | CCACATTTCCAACAGCCYYTT | 2019 | 2039 | 21 | 47.4 | 57.5-61.1 | - |  |
| SC_CON_9_2251_F | 1 | TCCCTCARATCACTCTTTGGCA | 2251 | 2272 | 22 | 47.6 | 59.9-61.1 | + | 363 |
| SC_CON_9_2614_R | 1 | TCTTCTGTYAATGGCCATTGTT | 2614 | 2635 | 21 | 38.1 | 56.2-58.5 | - |  |
| SC_CON_11_2862_F | 1 | ACAGTRCTRGATGTRGGDGAT | 2868 | 2889 | 22 | 50 | 53.7-64.6 | + | 378 |
| SC_CON_11_3245_R | 1 | TCCATTTRTCAGGATGGAGYTCA | 3245 | 3267 | 23 | 42.9 | 57.0-61.7 | - |  |
| SC_CON_13_3514_F | 1 | CAAAAGACTTAATAGCAGAAATACAGAAACA | 3514 | 3544 | 31 | 29 | 59.5 | + | 342 |
| SC_CON_13_3865_R | 1 | GCTGCYCCATCTACATAGAARG | 3865 | 3886 | 22 | 50 | 56.4-60.3 | - |  |
| SC_CON_15_4050_F | 1 | TATGCAYTAGGRATYATTCARGCAC | 4050 | 4074 | 25 | 38.1 | 56.1- 62.5 | + | 430 |
| SC_CON_15_4482_R | 1 | TCTGCTGGRATAACYTCTGCTTC | 4482 | 4504 | 23 | 47.6 | 58.7-62.0 | - |  |
| SC_CON_17_4749_F | 1 | ACAGCAGTACARATGGCAGTATTC | 4749 | 4772 | 24 | 43.5 | 59.6-60.7 | + | 331 |
| SC_CON_17_5077_R | 1 | CCATGTKYTAATCYTCATCCTGTCTAC | 5077 | 5103 | 27 | 41.7 | 58.4-62.8 | - |  |
| SC_CON_19_5326_F | 1 | ACACAARTAGAYCCTRRCCTRGC | 5326 | 5349 | 23 | 50 | 55.6-66.5 | + | 379 |
| SC_CON_19_5683_R | 1 | ACYCCTGCCCAAGTATCYCC | 5709 | 5728 | 20 | 61.1 | 58.5 - 63.5 | - |  |
| SC_CON_21_5970_F | 1 | ATGGCAGGAAGAAGMGGARAC | 5970 | 5990 | 21 | 52.6 | 57.9 - 62.5 | + | 365 |
| SC_CON_21_6333_R | 1 | ACAGGYACCCCATARTADACWGT | 6333 | 6355 | 23 | 45 | 56.0 - 62.8 | - |  |
| SC_CON_23_6579_F | 1 | TGTGTAAARYTRACCCCWCTYTGYGT | 6579 | 6604 | 26 | 42.9 | 59.8 - 68.1 | + | 369 |
| SC_CON_23_6953_R | 1 | ATYCCATGTGTRCAYTGTACTGWR | 6953 | 6973 | 24 | 40 | 55.9 - 63.1 | - |  |
| SC_CON_25_7193_F | 1 | AGGARAYATAAGRMAAGCAYATT | 7193 | 7215 | 23 | 27.8 | 49.7 - 60.3 | + | 325 |
| SC_CON_25_7521_R | 1 | ATGGGAGGGGCATAYATTG | 7522 | 7540 | 20 | 50 | 53.6 - 56.8 | - |  |
| SC_CON_27_7834_F | 1 | TGACGGTACAGGCYARACA | 7834 | 7852 | 19 | 52.9 | 56.0 - 60.5 | + | 391 |
| SC_CON_27_8221_R | 1 | AAACCAAYTCCACARAYTTKYCC | 8221 | 8243 | 23 | 38.9 | 55.0 - 63.4 | - |  |
| SC_CON_29_8510_F | 1 | CCTGTGCCTYTTCAGCTACC | 8510 | 8529 | 20 | 57.9 | 58.8 - 60.1 | + | 245 |
| SC_CON_29_8749_R | 1 | GCCYTGTCTKATTCTTSTAGGTATRT | 8749 | 8774 | 26 | 39.1 | 56.2 - 62.3 | - |  |
| SC_CON_31_8797_F | 1 | ATGGGDRGCAARTGGTCAAAA | 8797 | 8817 | 21 | 44.4 | 56.0 - 62.5 | + | 269 |
| SC_CON_31_9064_R | 1 | AGTCCCCCCTTTTCTTTTAAAAA | 9064 | 9086 | 23 | 34.8 | 56.4 | - |  |
| SC_CON_33_9080_F | 1 | GGGGACTGGAWGGGYTARTT | 9080 | 9099 | 20 | 55.6 | 56.8 - 61.0 | + | 318 |
| SC_CON_33_9394_R | 1 | TCAKCAGTYYTTGWARWACTCCGG | 9394 | 9417 | 24 | 45 | 58.4 - 64.4 | - |  |
| SC_CON_2_63_F | 2 | CAAGGYTWCTTCCCWGATTGGC | 63 | 84 | 22 | 54.5 | 61.3 - 61.8 | + | 412 |
| SC_CON_2_481_R | 2 | CAGAGAGCTCCCRGGC | 481 | 496 | 16 | 73.3 | 55.6 - 58.7 | - |  |
| SC_CON_4_672_F | 2 | CCAGAGRAGHTCTCTCGACGC | 672 | 692 | 22 | 63.2 | 60.0 - 64.3 | + | 402 |
| SC_CON_4_1070_R | 2 | TCTARRGCTTCYTTGGTGTCTYKTA | 1070 | 1094 | 25 | 40 | 56.9 - 64.6 | - |  |
| SC_CON_6_1307_F | 2 | CAGCATTATCAGAARGAGCCAC | 1307 | 1328 | 22 | 47.6 | 57.5 - 59.1 | + | 354 |
| SC_CON_6_1655_R | 2 | TCTACATARTCTCTRAARGGTTCYT | 1658 | 1682 | 25 | 33.3 | 53.5 - 60.0 | - |  |
| SC_CON_8_1963_F | 2 | TGTTTCAAYTGTGGCAARGARG | 1963 | 1984 | 22 | 42.1 | 56.3 - 61.3 | + | 372 |
| SC_CON_8_2331_R | 2 | TCTTCTAATACTGTRTCATCTGCTCC | 2331 | 2356 | 26 | 40 | 57.9 - 60.2 | - |  |
| SC_CON_10_2562_F | 2 | ATTGARACTGTACCAGTAAMATTAAA | 2562 | 2589 | 28 | 28 | 56.0 - 59.9 | + | 429 |
| SC_CON_10_2988_R | 2 | GTGAYCCTTTCCATCCYTGTGG | 2997 | 3018 | 22 | 55 | 59.0 - 62.9 | - |  |
| SC_CON_12_3198_F | 2 | CCAGAYAARAARCATCAGAAAGAACC | 3198 | 3223 | 26 | 39.1 | 57.3 - 62.2 | + | 362 |
| SC_CON_12_3557_R | 2 | ATRGYTCYTGRTWAATTTGATATGTCCAY | 3557 | 3585 | 29 | 29.2 | 56.1 - 63.5 | - |  |
| SC_CON_14_3789_F | 2 | CCTGARTGGGARTTTGTYAATACY | 3783 | 3806 | 24 | 40 | 55.1 - 61.1 | + | 389 |
| SC_CON_14_4171_R | 2 | TCTACYTGTTCATTTCCTCCAATYC | 4171 | 4195 | 25 | 39.1 | 57.3 - 60.3 | - |  |
| SC_CON_16_4389_F | 2 | GTAGAYTGYAGTCCAGGRATATGG | 4389 | 4412 | 24 | 47.6 | 56.2 - 62.1 | + | 386 |
| SC_CON_16_4771_R | 2 | CCCCCTTTTCTTTTAAAATTGTGRATGA | 4771 | 4798 | 28 | 33.3 | 59.5 - 60.9 | - |  |
| SC_CON_18_4956_F | 2 | TGGAAAGGTGAAGGGGCAGT | 4956 | 4975 | 20 | 55 | 61.4 | + | 481 |
| SC_CON_18_5434_R | 2 | TGTCCTGCTKGATADTYACAMCT | 5434 | 5456 | 23 | 39.1 | 55.9 - 63.0 | - |  |
| SC_CON_20_5643_F | 2 | GAAGCWGTYAGACAYTTTCCTAGR | 5643 | 5666 | 24 | 45.83 | 56.5 - 60.9 | + | 324 |
| SC_CON_20_5970_R | 2 | GYYTCCKCTTCTTCCTGCCAT | 5970 | 5990 | 21 | 55.6 | 57.9 - 65.1 | - |  |
| SC_CON_22_6206_F | 2 | AGAGCAGAAGAYAGTGGMAATGA | 6206 | 6228 | 23 | 50 | 57.1 - 62.1 | + | 355 |
| SC_CON_22_6560_R | 2 | ACACATGGYTTTAGRCTTTSATCC | 6560 | 6583 | 24 | 40.9 | 57.4 - 61.7 | - |  |
| SC_CON_24_6858_F | 2 | CCAATTCCYATACATTAYTGTRCYCC | 6858 | 6883 | 26 | 40.9 | 55.5 - 63.8 | + | 454 |
| SC_CON_24_7316_R | 2 | AAYTTCTRRGTCCCCTCCTGAG | 7316 | 7337 | 22 | 52.6 | 56.9 - 63.1 | - |  |
| SC_CON_26_7482_F | 2 | ATAAARCAAATTRTAARHATGTGGC | 7482 | 7506 | 27 | 23.8 | 51.0 - 59.4 | + | 433 |
| SC_CON_26_7921_R | 2 | CCCCAGACYGTGAGYTKCA | 7921 | 7939 | 19 | 62.5 | 56.6 - 64.5 | - |  |
| SC_CON_28_8016_F | 2 | TGCTCTGGAAAACWCATYTGC | 8016 | 8036 | 21 | 45 | 57.4 - 59.1 | + | 508 |
| SC_CON_28_8522_R | 2 | AGTCTCTCAAKYGGTGGTAGCTG | 8522 | 8544 | 23 | 52.4 | 60.1 - 64.1 | - |  |
| SC_CON_30_8751_F | 2 | ATACCYASAAGRATMAGACARGGC | 8751 | 8774 | 24 | 45 | 55.8 - 64.0 | + | 318 |
| SC_CON_30_9074_R | 2 | CCWTCCAGTCCCCCCTTTT | 9074 | 9092 | 19 | 57.9 | 58.8 - 58.9 | - |  |
| SC_CON_32_9390_F | 2 | CAAGGYTWCTTCCCWGATTGGC | 9147 | 9167 | 20 | 54.5 | 61.3 - 61.8 | + | 243 |
| SC_CON_32_9390_R | 2 | TYYTTGWAGWACTCCGGATGY | 9390 | 9410 | 21 | 44.4 | 54.6 - 60.1 | - |  |

All primer pairs designed in the study. Primers in pool 1 starts with primer-pair #3. Primers were designed to cover bases 488-9389 of the HXB2 reference genome. Primer #2 & #32 Forward have the same sequence.

**Supplement table 2. HIV-1 FASTA accession numbers used to design the primers used in this study (Supp. Table 1)**

**Supplement table 2.1: Training dataset**

| **FASTA sequence accession number** |  |
| --- | --- |
| 01_AE.CN.2009.ZK052.JX112869 | |
| 02_AG.CM.2007.CM100-17.KU168310 | |
| 07_BC.TW.2013.pCRF07.KF234628 | |
| 08_BC.CN.2007.2007CNGX_HK.JF719819 | |
| 13_cpx.CM.2006.363-24.MH705141 | |
| 27_cpx.FR.2004.04CD_FR_KZS.AM851091 | |
| 30_0206.GH.2003.03GH195AG_06.AB286854 | |
| 45_cpx.FR.2004.04FR-AUK.EU448295 | |
| 60_BC.FR.2006.06FR-CRN.EU448296 | |
| 60_BC.IT.2011.BAV499.KC899079 | |
| 60_BC.IT.2011.BAV514.KC899080 | |
| 60_BC.IT.2011.BAV636.KC899081 | |
| A1.UG.2007.p191084.JX236669 | |
| A1.UG.2007.p191845.JX236671 | |
| A1.UG.2007.p9004SDM.JX236676 | |
| A1.RW.2007.pR463F.JX236677 | |
| A1.RW.2007.pR880F.JX236678 | |
| A1.CM.2003.CM54-7.KU168305 | |
| A1.TZ.2008.DEMA108TZ002.KY658694 | |
| A1.TZ.2008.DEMA108TZ004.KY658695 | |
| A1.PK.2014.DEMA114PK027.KY658714 | |
| A6.UA.2012.DEMA112UA024.KU749403 | |
| A6.UA.2012.DEMA112UA030.KU749404 | |
| A6.UA.2012.DEMA112UA034.KU749405 | |
| D.UG.2005.p190049.JX236668 | |
| D.UG.2007.p191647.JX236670 | |
| D.UG.2008.p191859.JX236672 | |
| D.UG.2007.p191882.JX236673 | |
| D.UG.2007.pSC191727.JX236679 | |
| D.CD.2003.LA17MuBo.KU168271 | |
| D.UG.2007.192002.MW006058 | |
| D.UG.2011.194535.MW006073 | |
| D.UG.2009.275031.MW006081 | |
| F1.RO.2003.LA20DuCl.KU168274 | |
| F1.-.2003.LA21LeAn.KU168275 | |
| G.GH.2003.GHNJ175.AB231893 | |
| G.GH.2003.03GH175G.AB287003 | |
| G.GH.2003.03GH175G.AB287004 | |
| G.CD.2003.LA23LiEd.KU168277 | |
| G.CM.2003.CM44-10.KU168302 | |
| G.CM.2005.144-26.MH705145 | |
| H.CD.2004.LA19KoSa.KU168273 | |
| B.US.2009.N-2.GU733717 | |
| B.US.2006.CH106_TF1.JN944897 | |
| B.US.2006.CH40_TF1.JN944905 | |
| B.US.2011.AMBI-CLONE.KU641402 | |
| B.AR.2014.DEMB14AR003.KY658685 | |
| B.PH.2015.DEMB15PH003.KY658690 | |
| B.-.2010.DEURF10HA002.KY658703 | |
| B.HT.2011.DEURF11HT001.MH078551 | |
| B.US.2013.ARC-1a.MK214316 | |
| B.BE.2017.02006_cen.MN449474 | |
| B.BE.2017.02006_chr10_sc.MN449475 | |
| B.BE.2017.02006.MN449476 | |
| B.PE.2007.SEC11A.MN887110 | |
| B.US.2015.C02.02_20181218CADHFH_MATR3.MT744340 | |
| B.US.2015.C03.02_20181029CADHFH_ZNF268.MT744356 | |
| B.US.2015.F07.02_20190325CADHFH_ZNF721.MT745572 | |
| B.US.2015.R09.02_20190805CADHFH_ABCA11P.MT745577 | |
| B.US.2019.P3_cmv_fbxo22.MW309889 | |
| C.IN.2003.D24.EF469243 | |
| C.MW.2007.703010131_CH131_TF.KC156114 | |
| C.ZA.2008.705010185_CH185.mo6.KC156116 | |
| C.ZA.2007.705010067_CH067_TF.KC156125 | |
| C.ZA.2007.705010162_CH162_TF.KC156126 | |
| C.IN.2009.T125_2139.KC156210 | |
| C.MW.2009.703010256_CH256.w96.KC156214 | |
| C.MW.2008.703010269_CH269.w24.KC156215 | |
| C.MW.2008.702010293_CH293.w8a.KC156216 | |
| C.MW.2008.702010293_CH293.w8b.KC156217 | |
| C.MW.2008.702010432_CH432.w4.KC156218 | |
| C.MW.2008.702010440_CH440.w4.KC156219 | |
| C.TZ.2008.707010457_CH457.w8.KC156220 | |
| C.ZA.2008.705010534_CH534.w12.KC156221 | |
| C.ZA.2007.CH236_TF.MN202472 | |
| U.NG.2011.DEURF11NG011.MH078559 | |

**Supplement table 2.2: Testing dataset**

| **FASTA sequence accession number** |  |  |
| --- | --- | --- |
| 01_AE.CN.2009.ZK052.JX112869 | | |
| 02_AG.-.2000.LA11ZaCh.KU168266 | |  |
| 02_AG.CM.2003.CM1193-8.KU168304 | |  |
| 01_AE.JP.2000.DR1873.AB253656 | |  |
| 03_A6B.BY.2000.98BY10443.AF414006 | |  |
| 08_BC.CN.2000.p00CH-WS035_08_BC51.AB746344 | |  |
| 08_BC.CN.2000.p00CH-WS035_08_BC52.AB746345 | |  |
| 08_BC.CN.2000.p00CH-HH090_08_BC02.AB773884 | |  |
| 08_BC.CN.2000.p00CH-HH090_08_BC30.AB773885 | |  |
| 08_BC.CN.2007.2007CNGX_HK.JF719819 | |  |
| 08_BC.CN.2000.QJ001.KC914396 | |  |
| 09_cpx.CI.2000.00IC_10092.AJ866553 | |  |
| 26_A5U.CD.2002.02CD_LBTB084.FM877781 | |  |
| 26_A5U.CD.2002.02CD_MBTB047.FM877782 | |  |
| 92_C2U.CD.2002.CG-0151-02V_NGSID1.KY392767 | |  |
| A1.CD.2002.LA01AlPr.KU168256 | |  |
| B.-.2002.LA04GuFu.KU168259 | |  |
| B.CN.2002.02HNsmx2.DQ007901 | |  |
| B.CN.2002.02HNsq4.DQ007902 | |  |
| B.CN.2002.02HNsc11.DQ007903 | |  |
| B.US.2001.pREJO_c.JN944943 | |  |
| B.JP.2001.DR388.AB289589 | |  |
| B.JP.2001.DR388.AB289590 | |  |
| B.CN.2001.01CN-DH001.AB750367 | |  |
| B.US.2001.REJO_TF1.JN944911 | |  |
| B.US.2000.RHPA_TF1.JN944917 | |  |
| B.US.2000.THRO_TF1.JN944930 | |  |
| B.US.2001.TRJO_TF1.JN944936 | |  |
| B.US.2000.WITO_TF1.JN944938 | |  |
| C.ZA.2001.01ZATM45.AY228557 | |  |
| C.ZM.2002.02ZM112.AB254143 | |  |
| C.ZM.2002.02ZM112.AB254145 | |  |
| C.ZM.2002.02ZM114.AB254146 | |  |
| C.ZM.2002.02ZM114.AB254147 | |  |
| C.ZM.2002.02ZM115.AB254148 | |  |
| C.ZM.2002.02ZMBC.AB254149 | |  |
| C.ZM.2002.02ZMDB.AB254150 | |  |
| C.ZM.2002.02ZMDB.AB254151 | |  |
| C.ZM.2002.02ZMDB.AB254152 | |  |
| C.ZM.2002.02ZMDB.AB254153 | |  |
| C.ZM.2002.02ZMDB.AB254154 | |  |
| C.ZM.2002.02ZMJC.AB254155 | |  |
| C.ZM.2002.02ZMJM.AB254156 | |  |


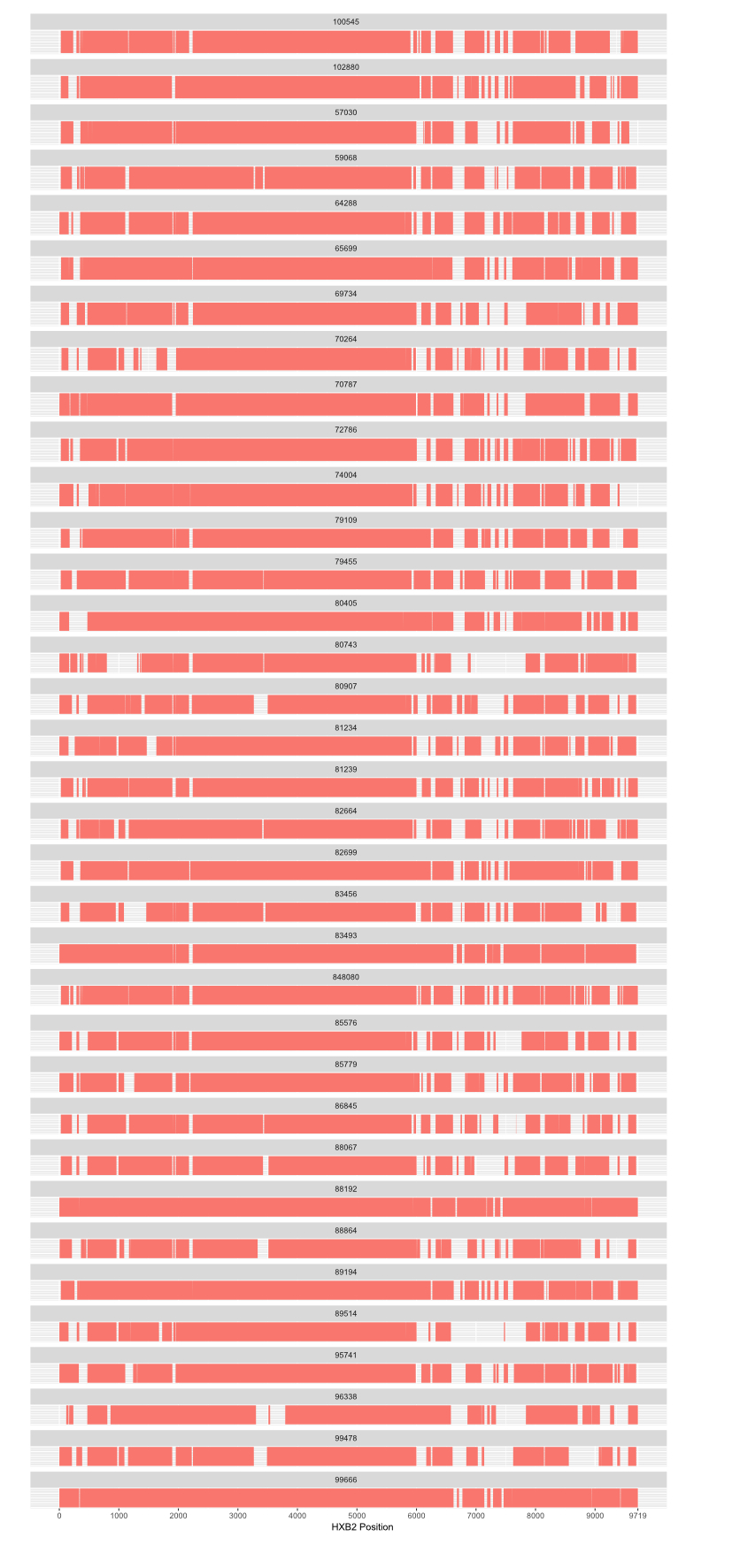


**Supplement 3. Coverage of the HXB2 reference genome bases across all samples HIV-1 NFLG sequences generated with the newly designed amplicons method presented in this study.**

The X-axis represents position in base-pairs (0-9719) of the HIV-1 reference genome (HXB2: K03455.1). The Y-axis is a binary axis (0 or 1), in which every position that had >5 counts coverage is represented by a full red bar (y=1). Position with coverage of <5 counts are represented by an empty space (y=0).


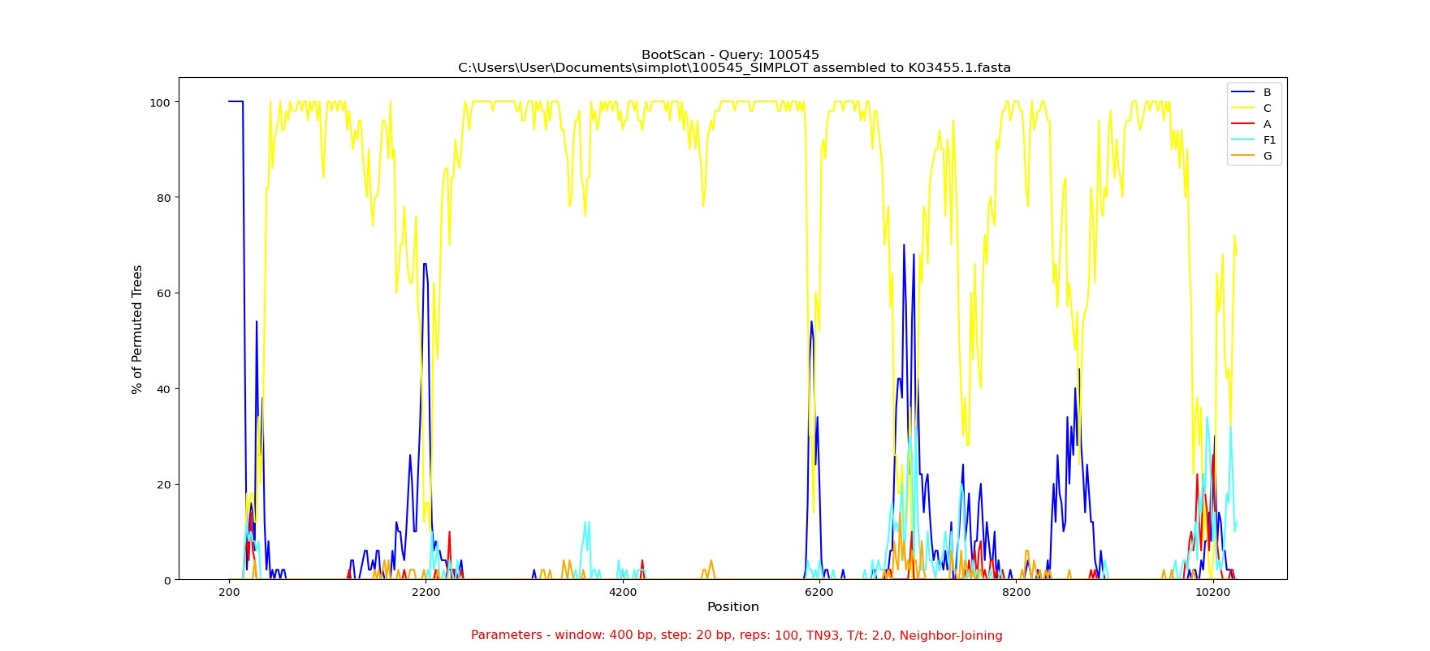


A


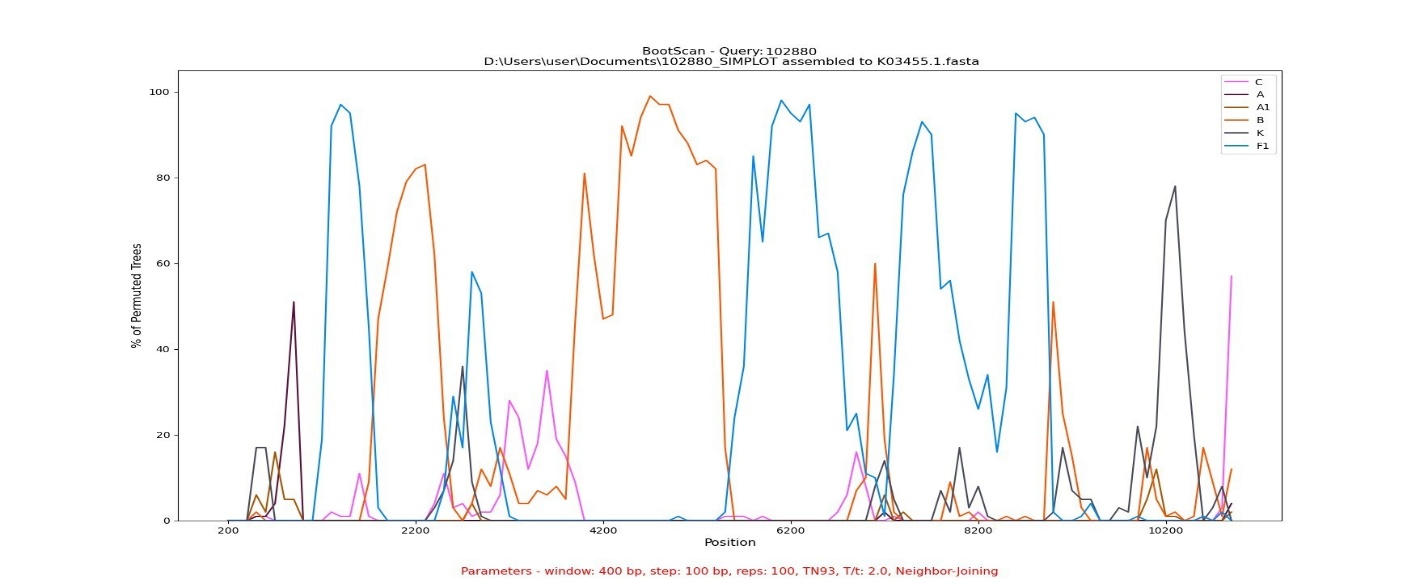


B


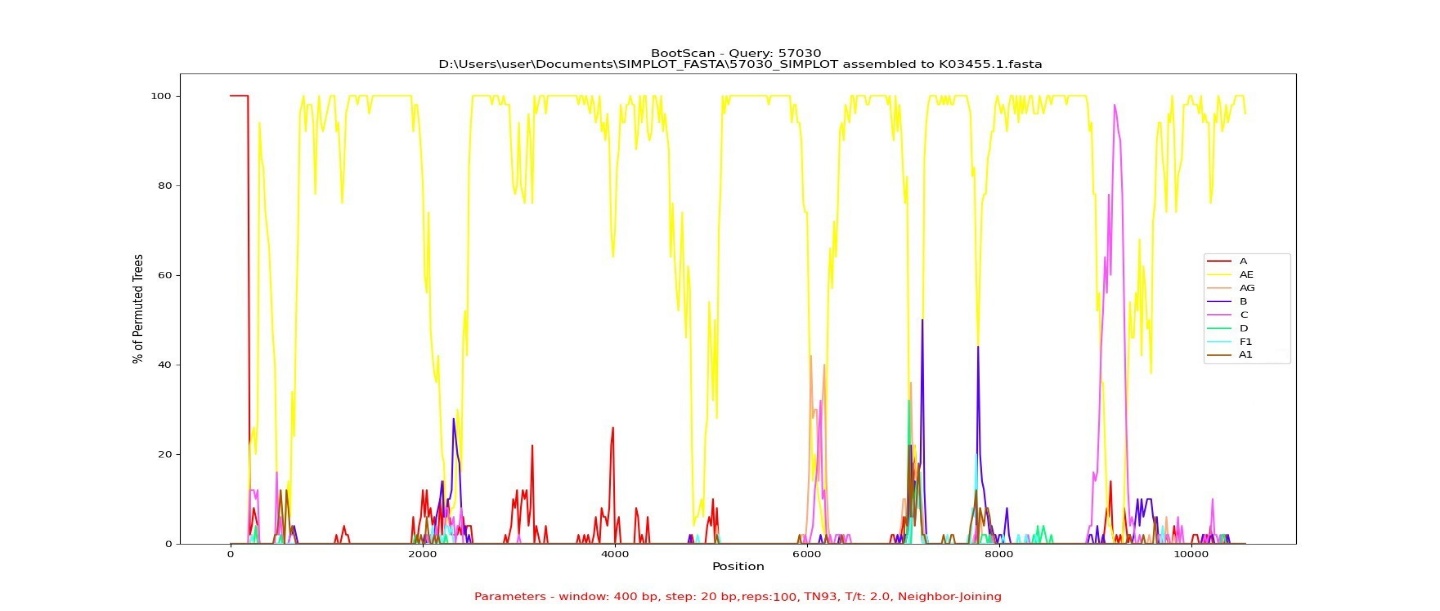


C
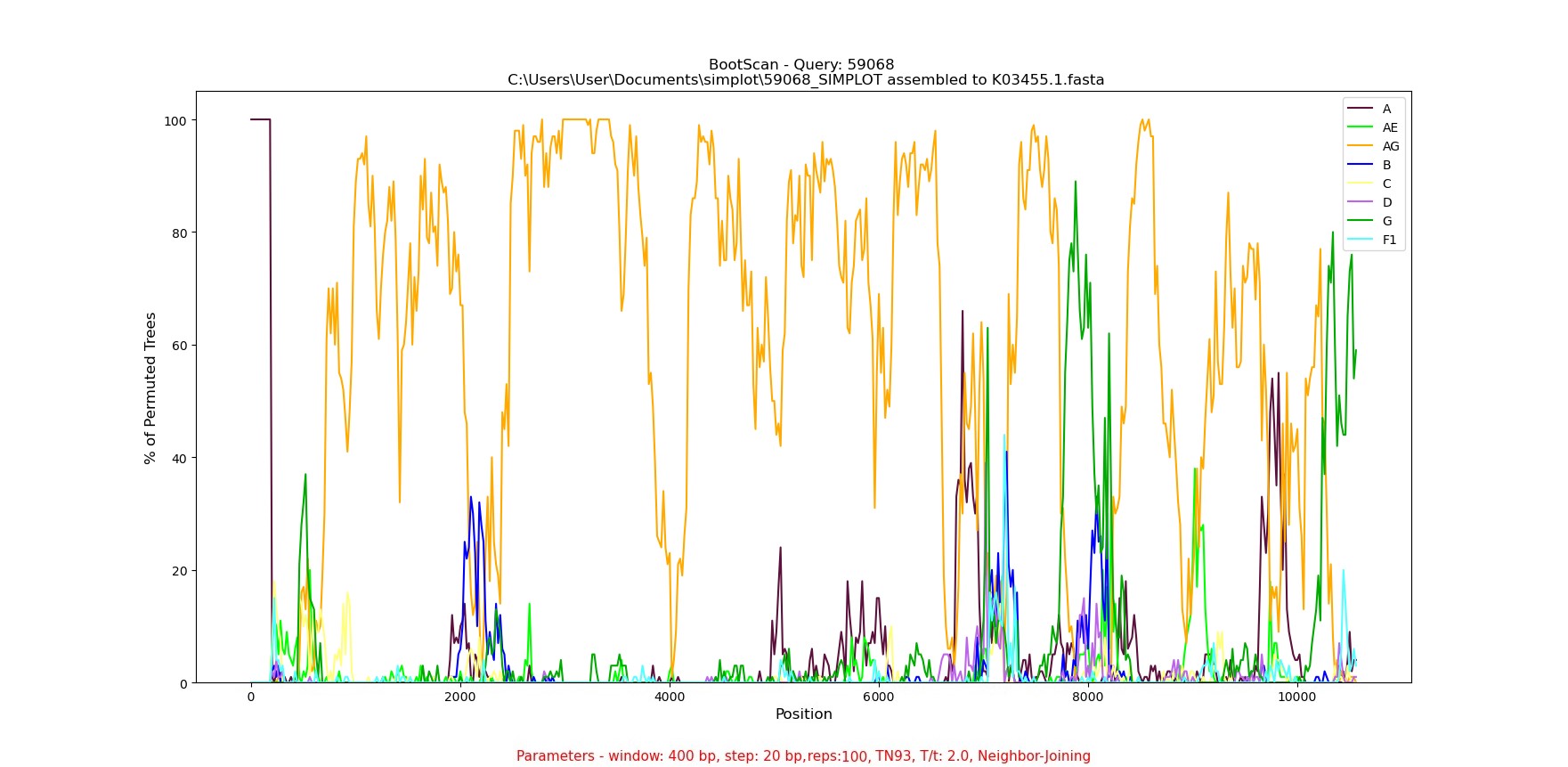


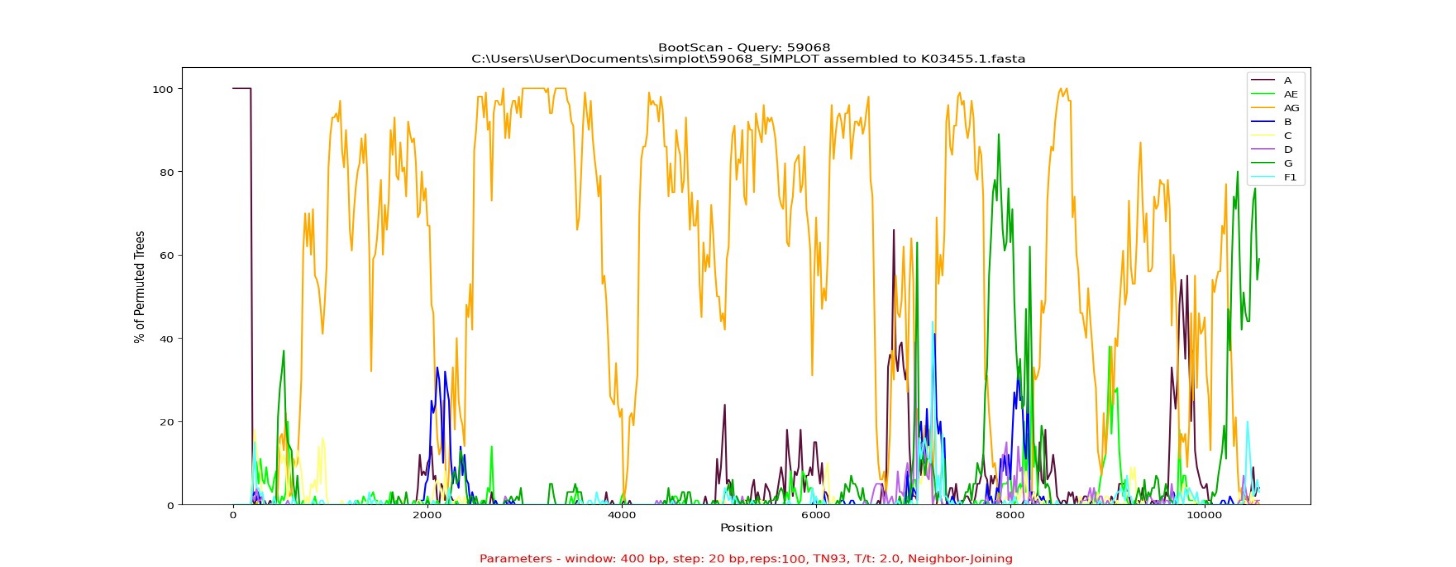


D
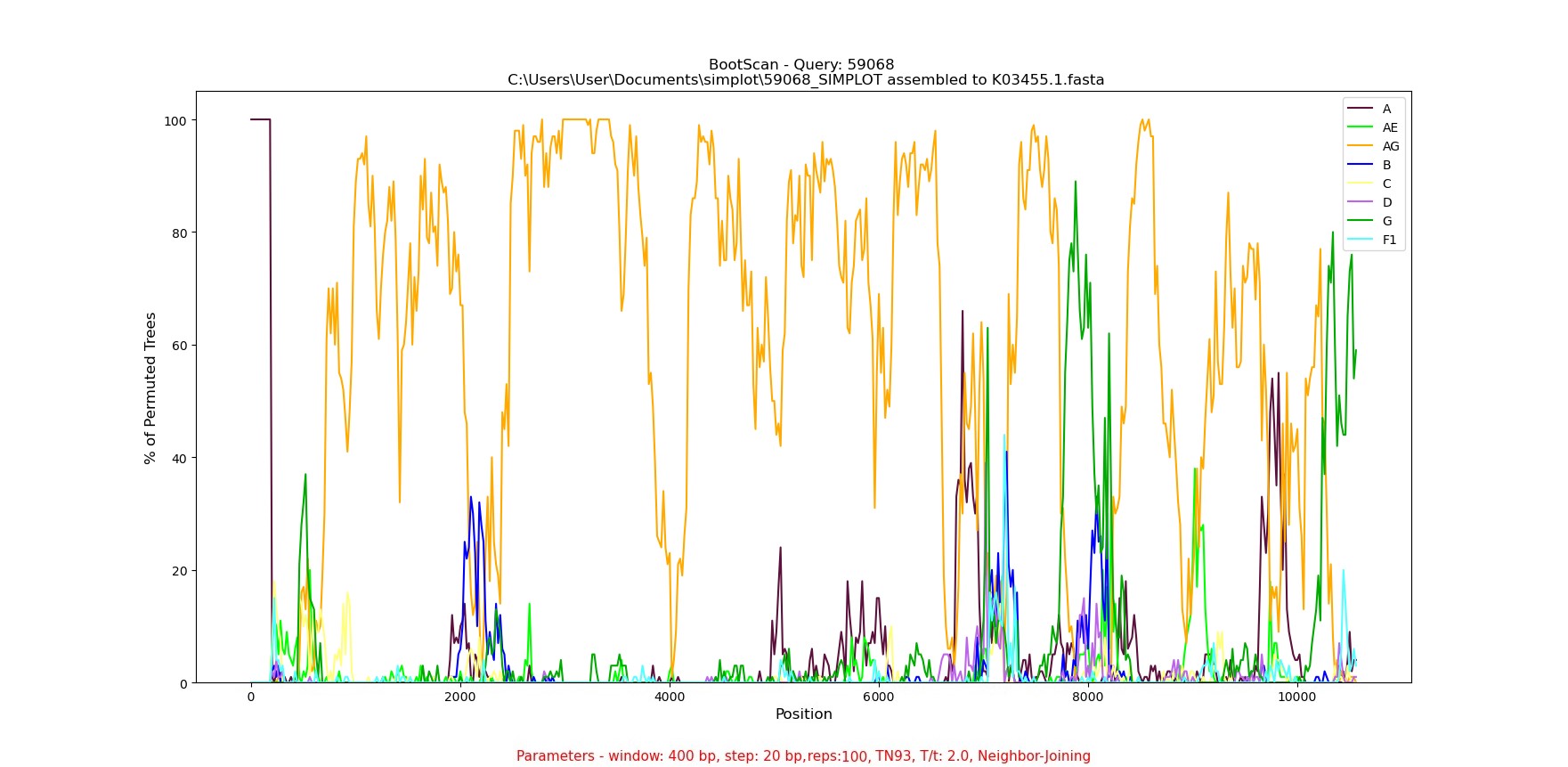


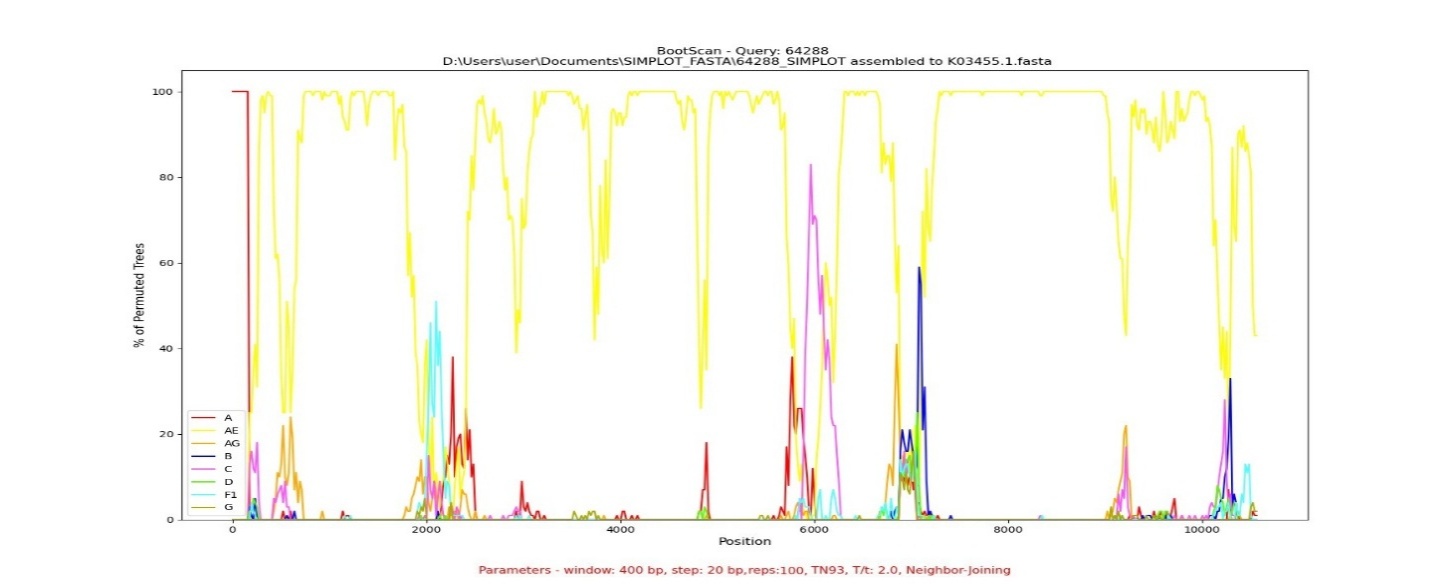


E


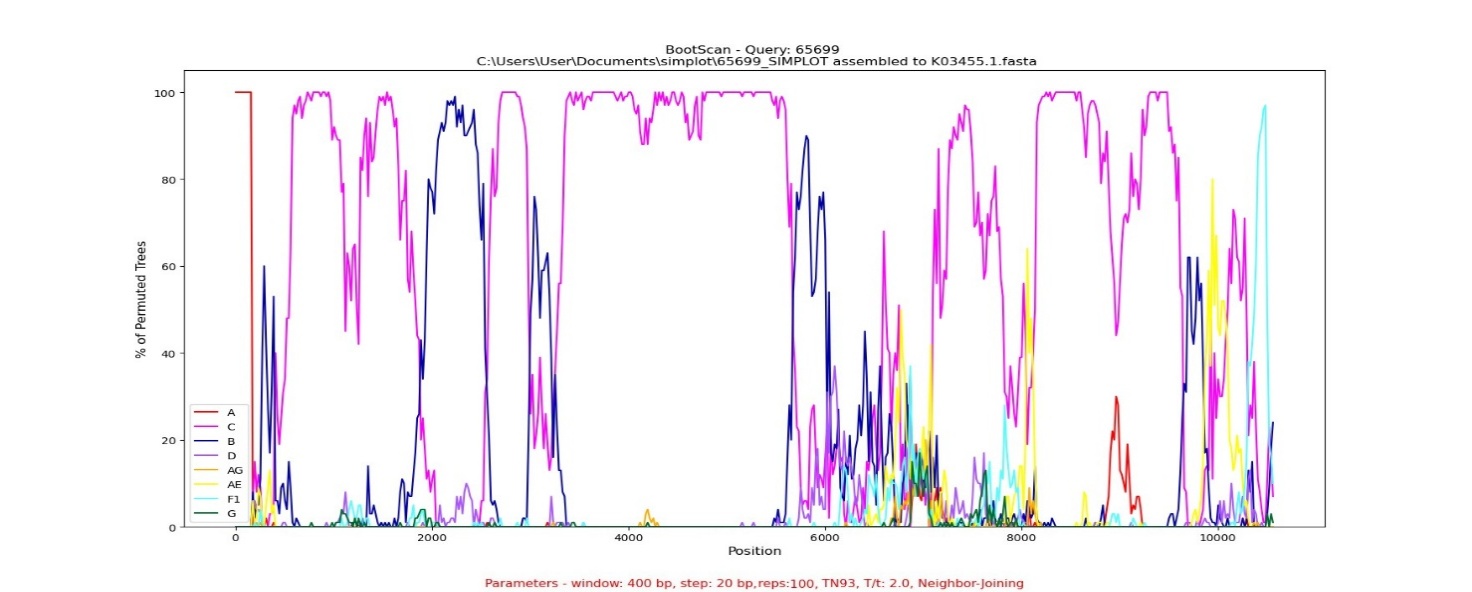


F


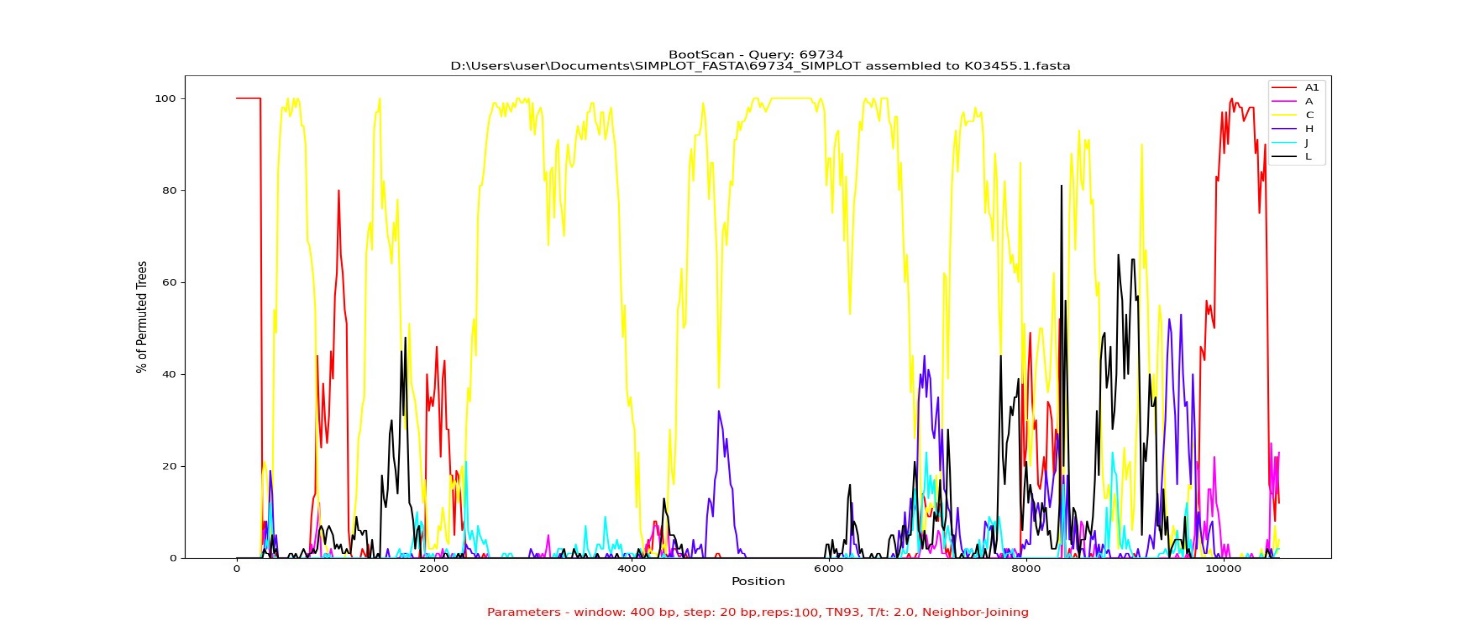


G


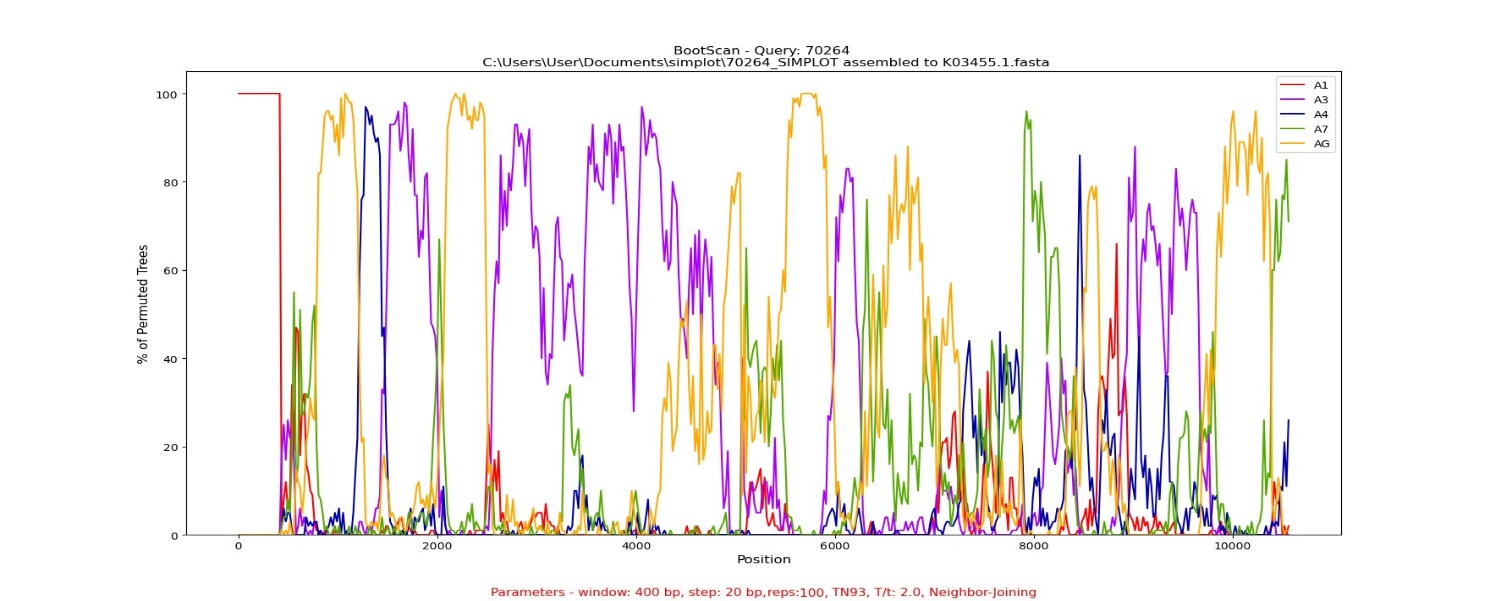


H


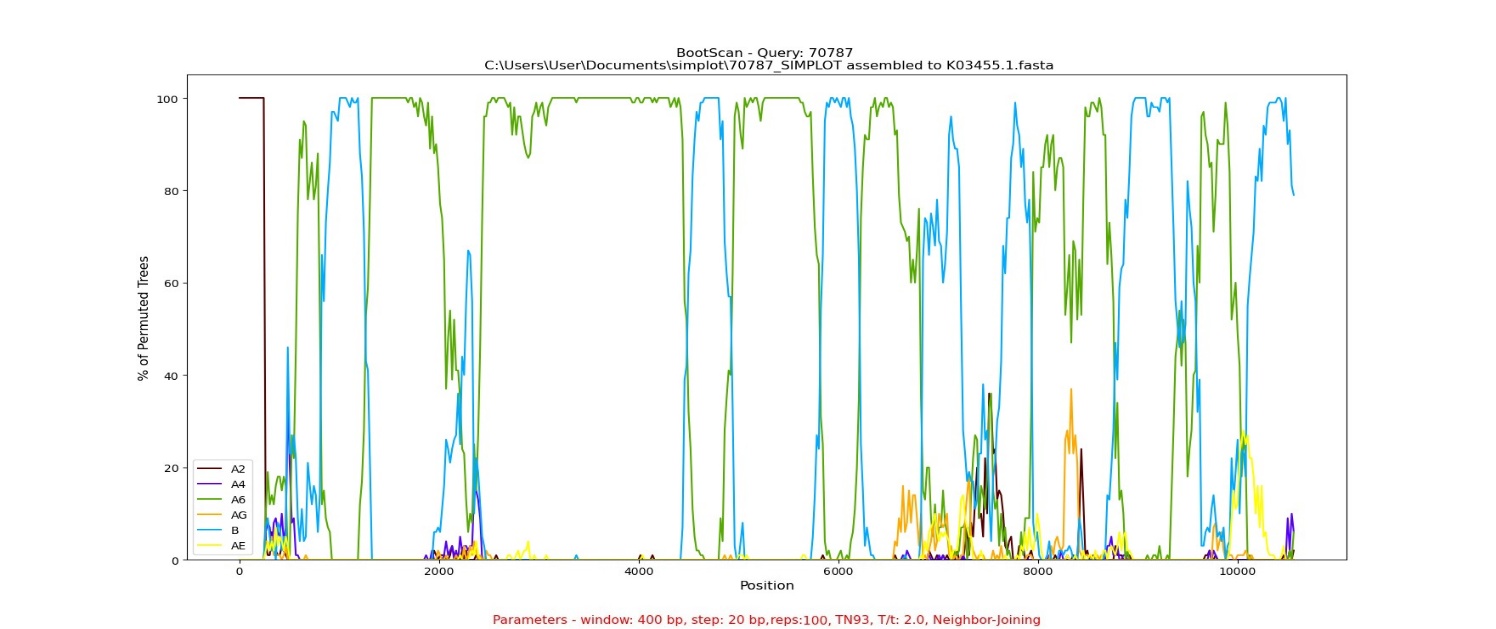


I


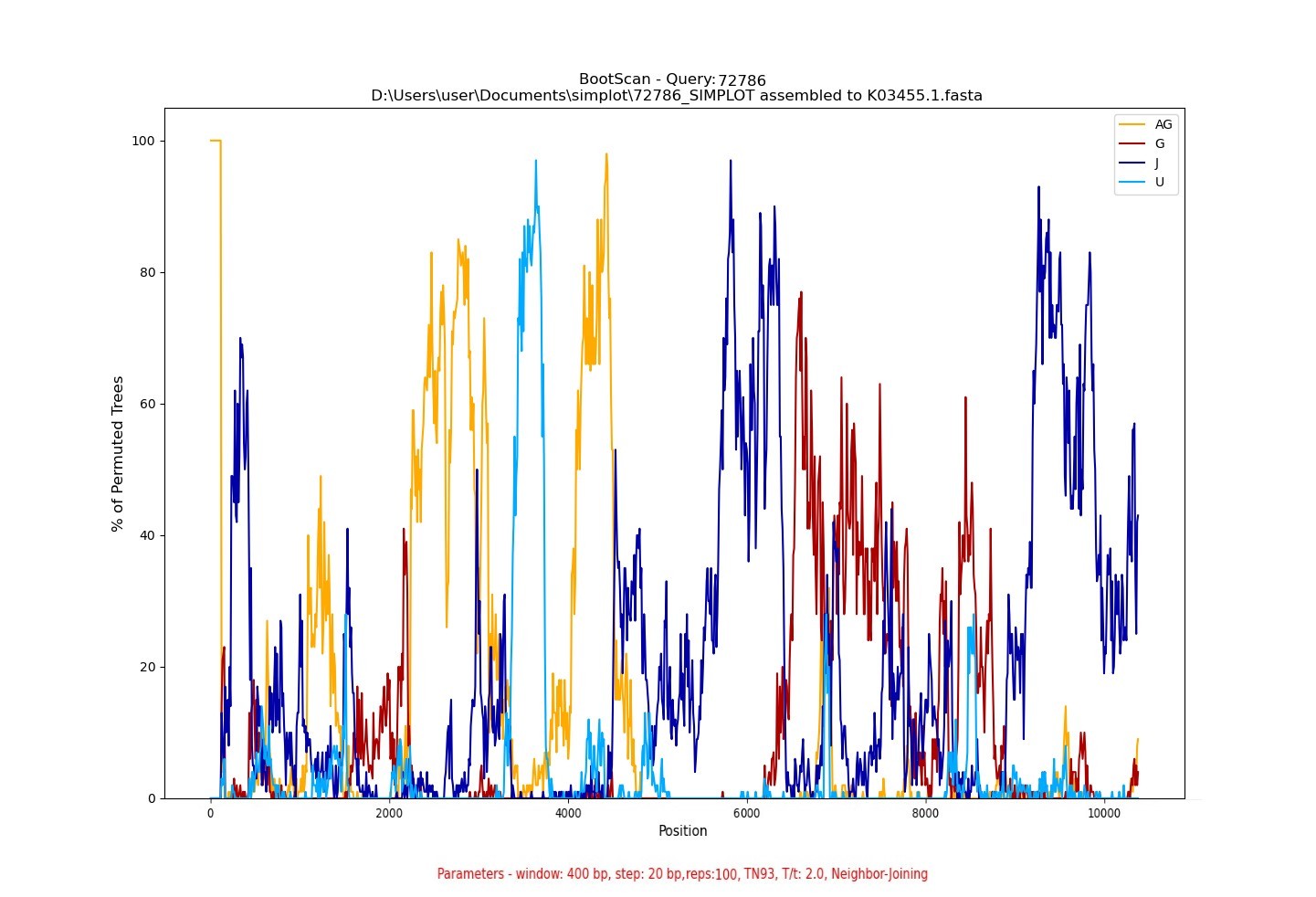


J


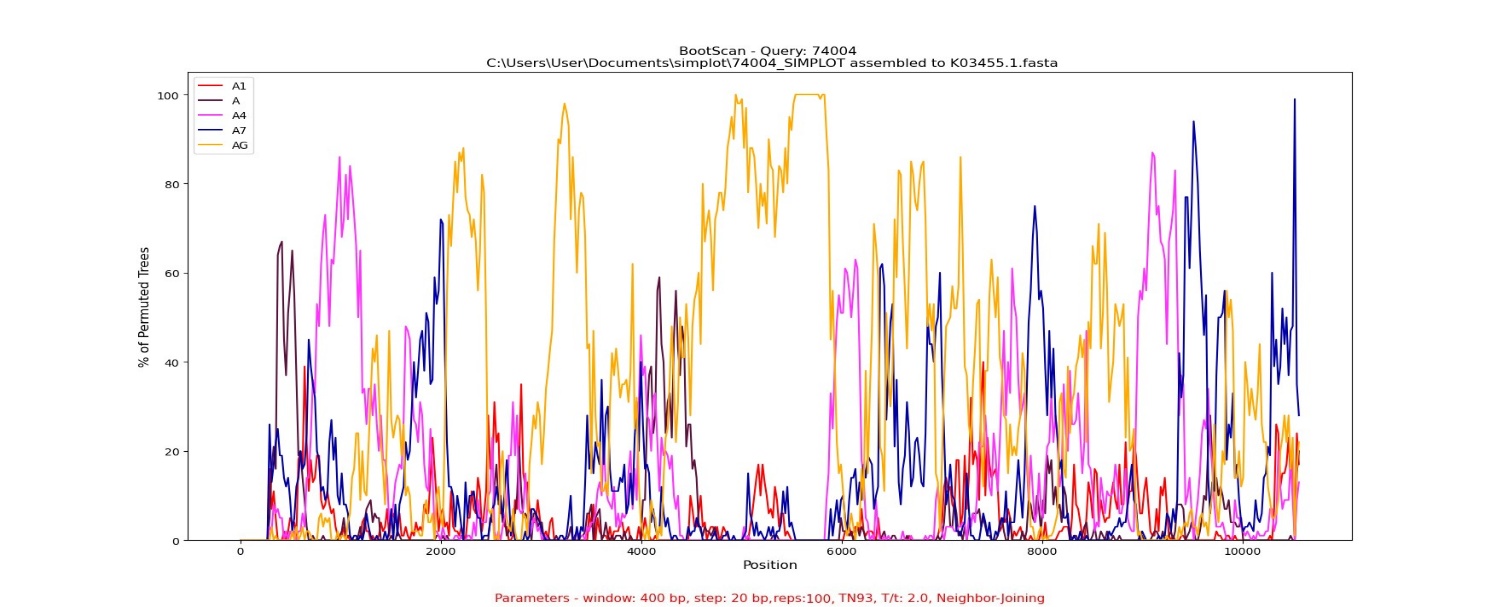


K


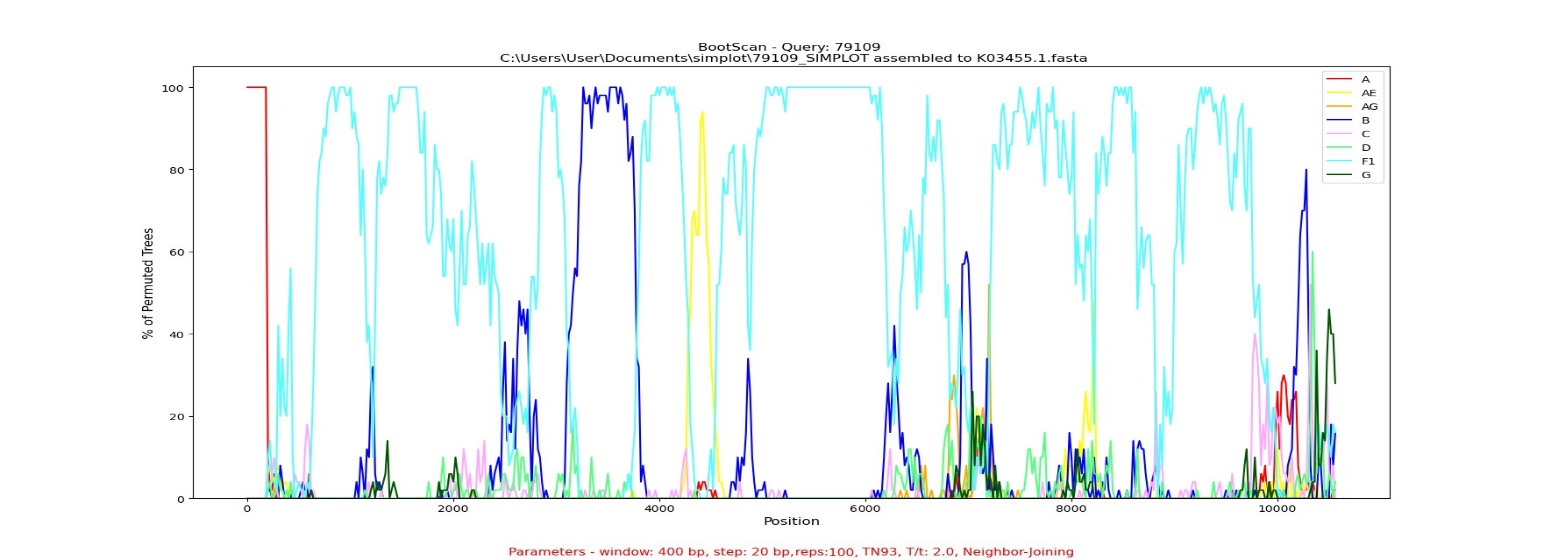


L


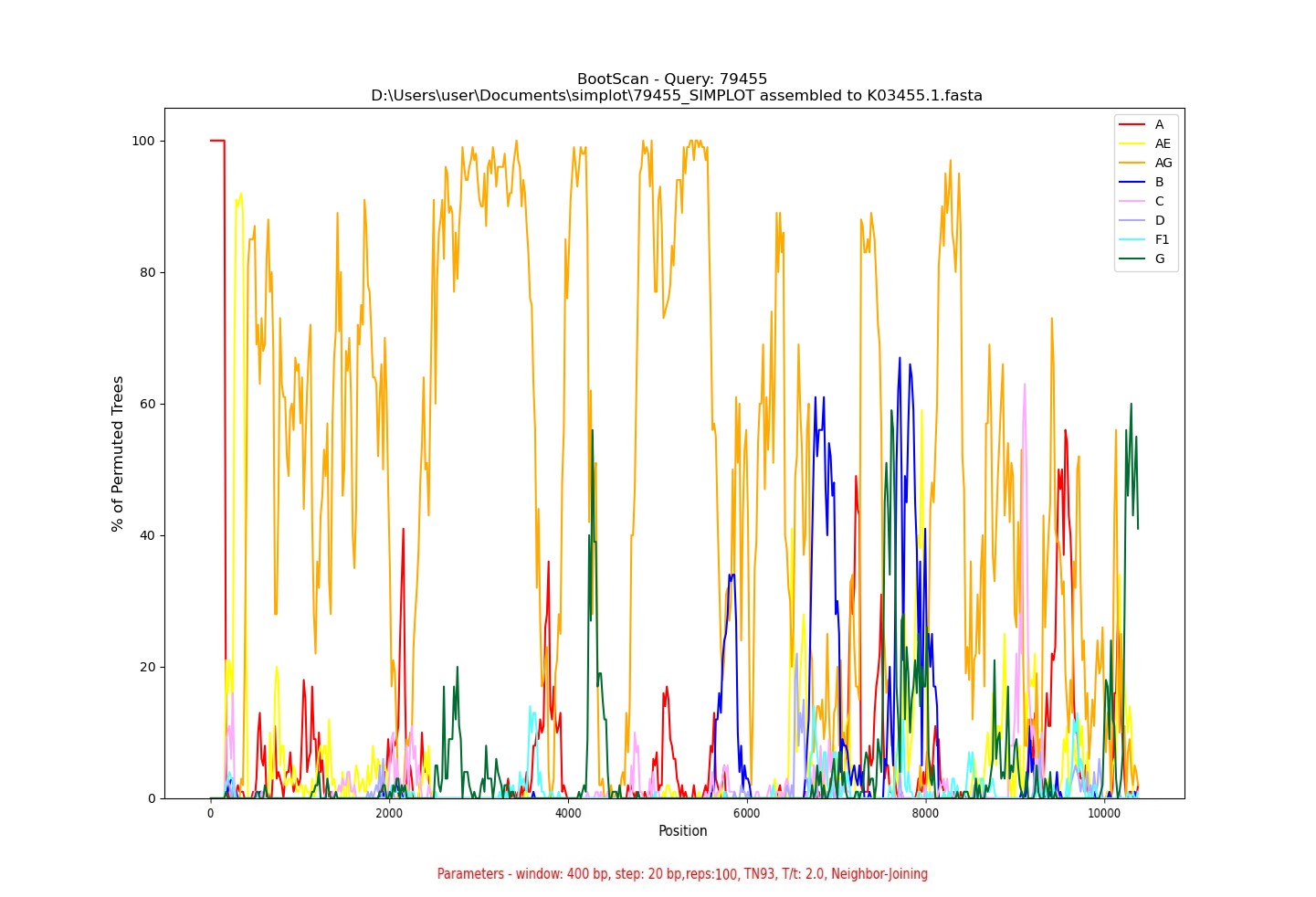


M


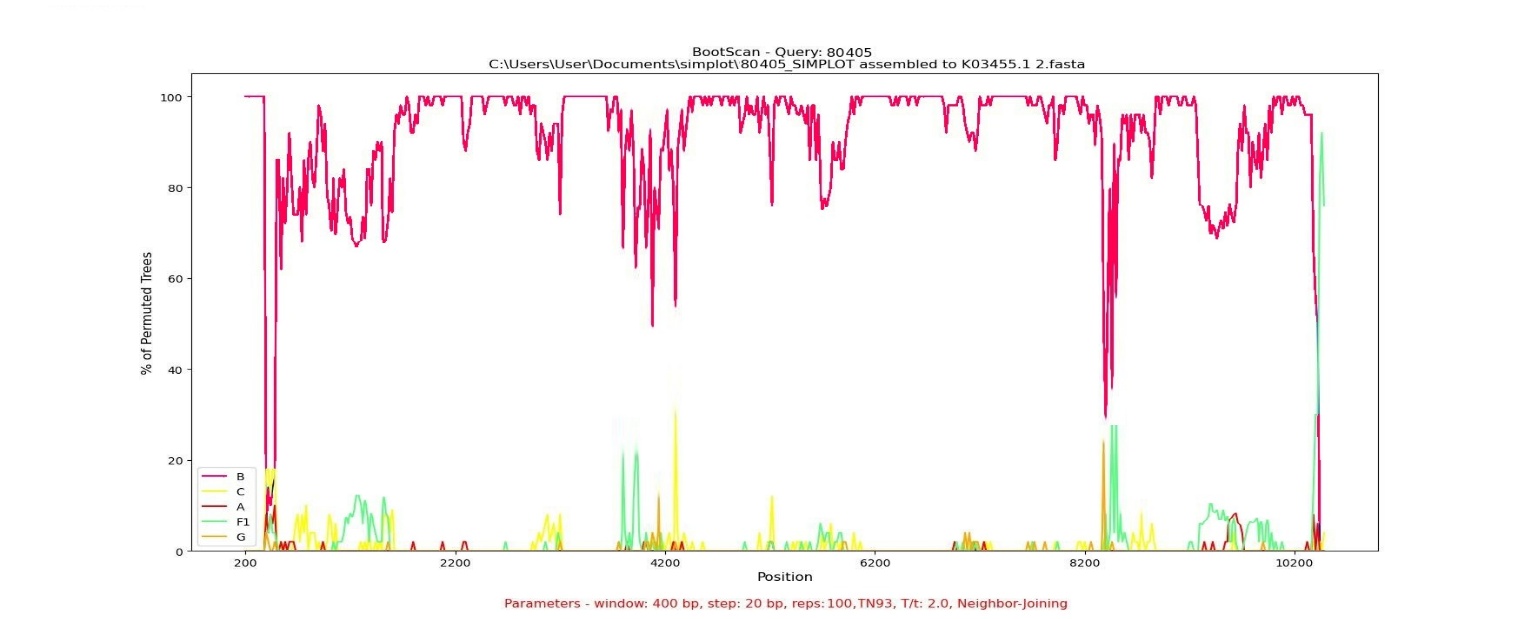


N


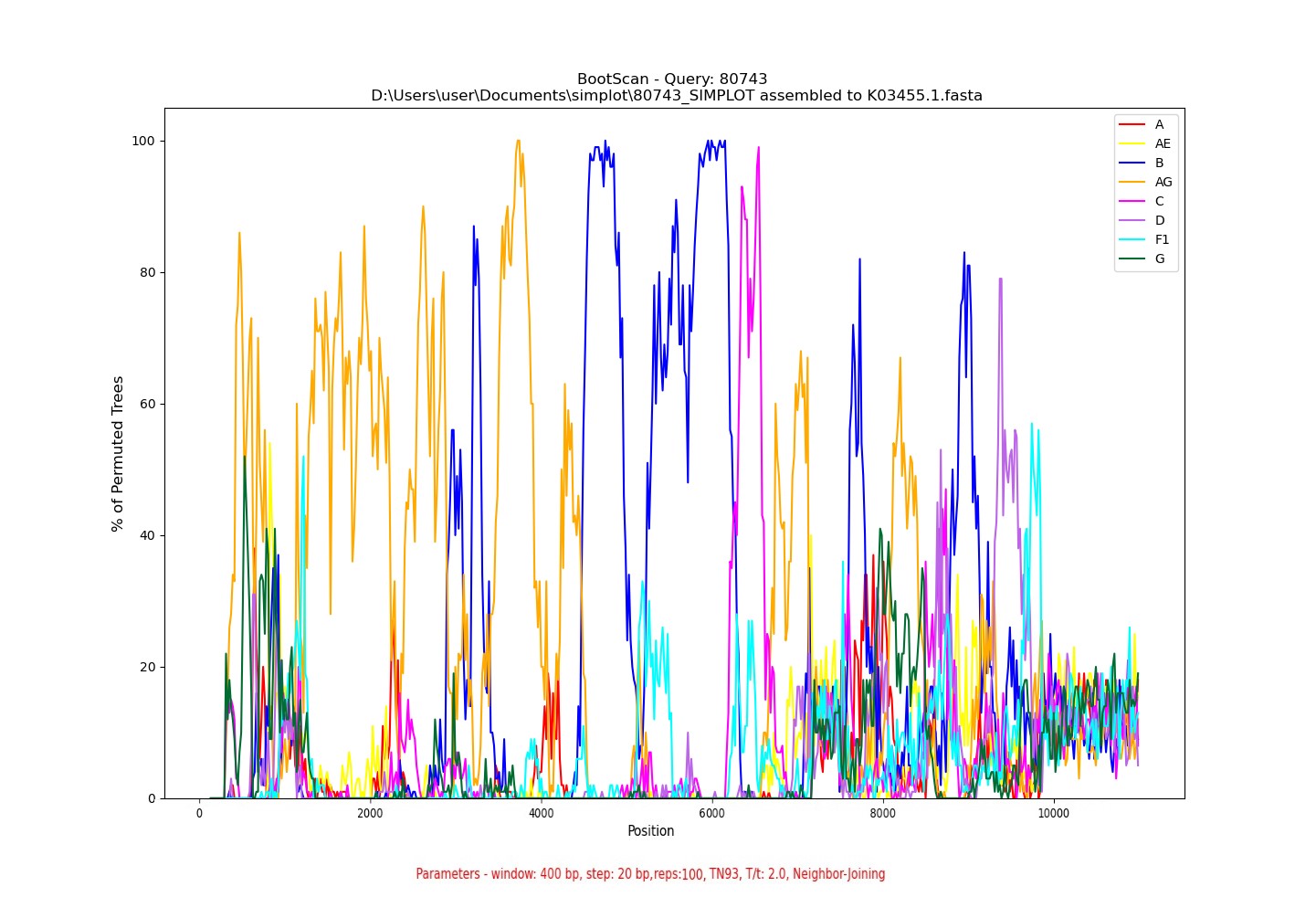


O


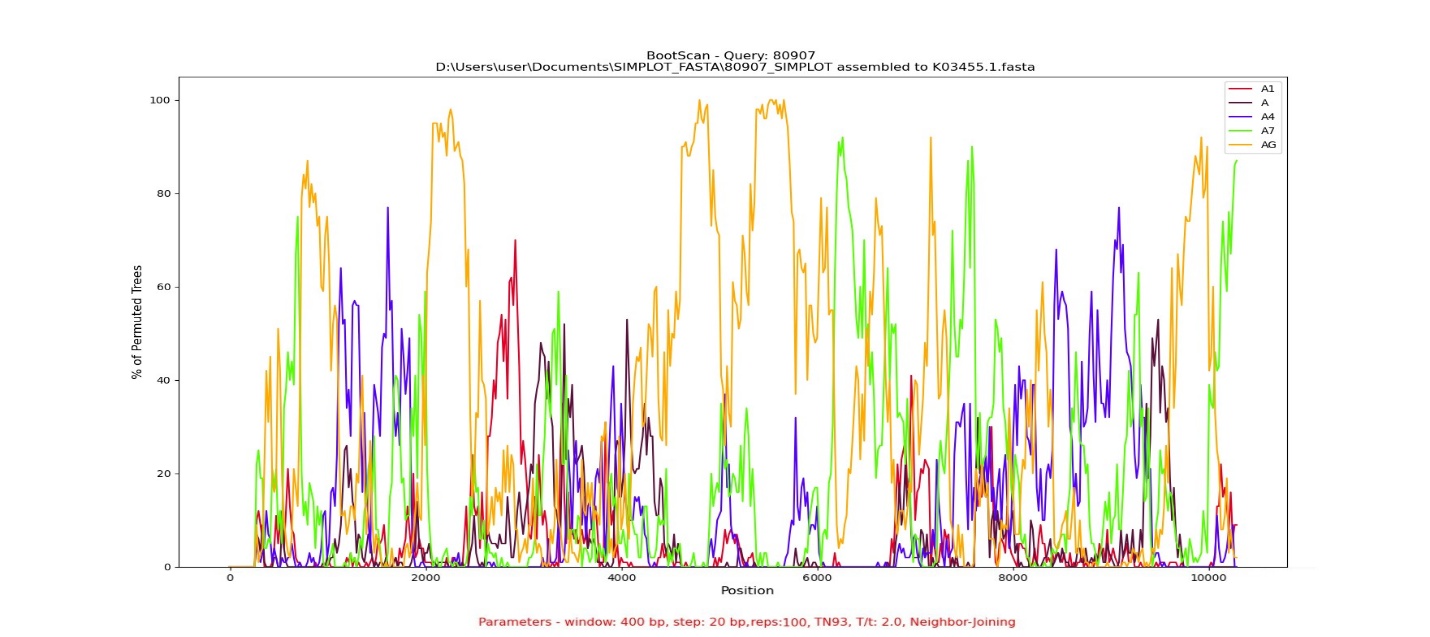


P


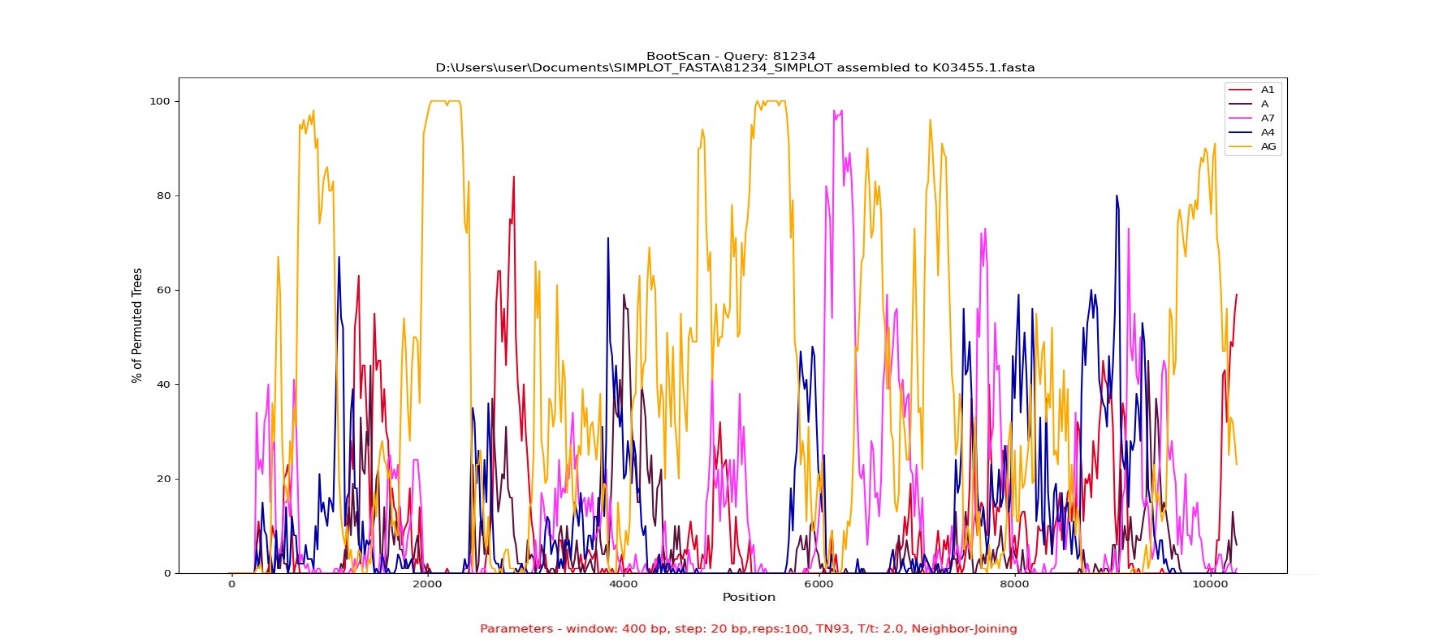


Q


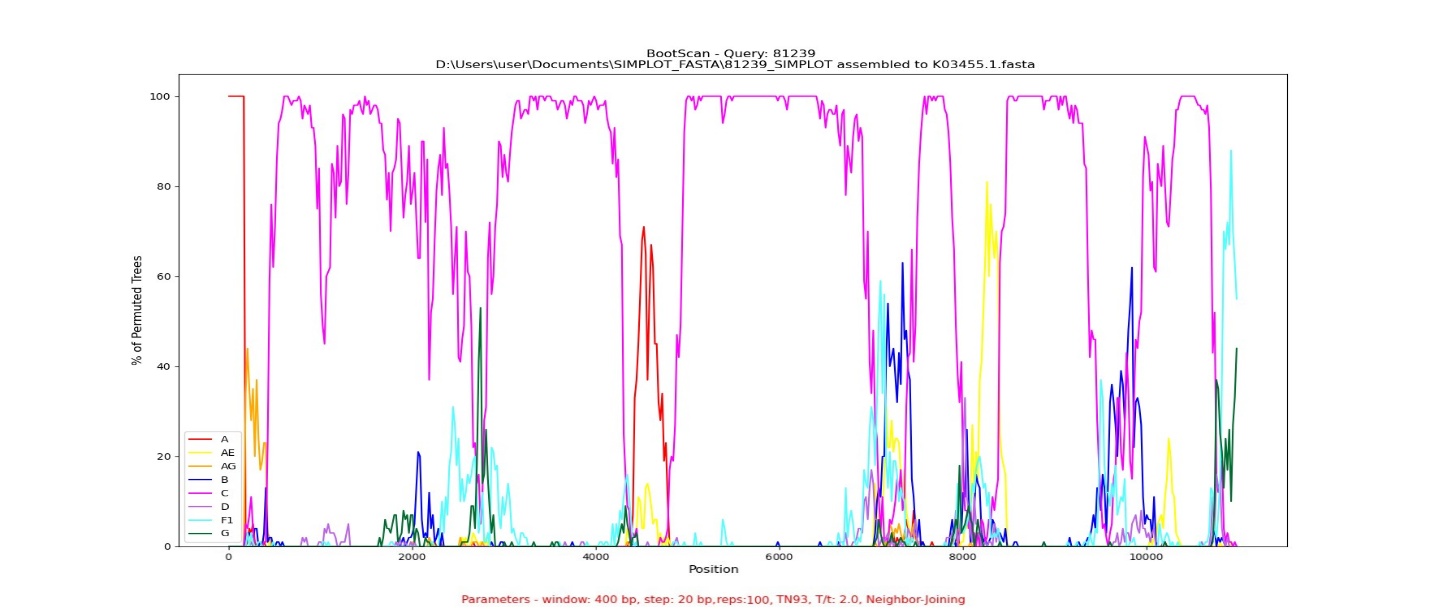


R


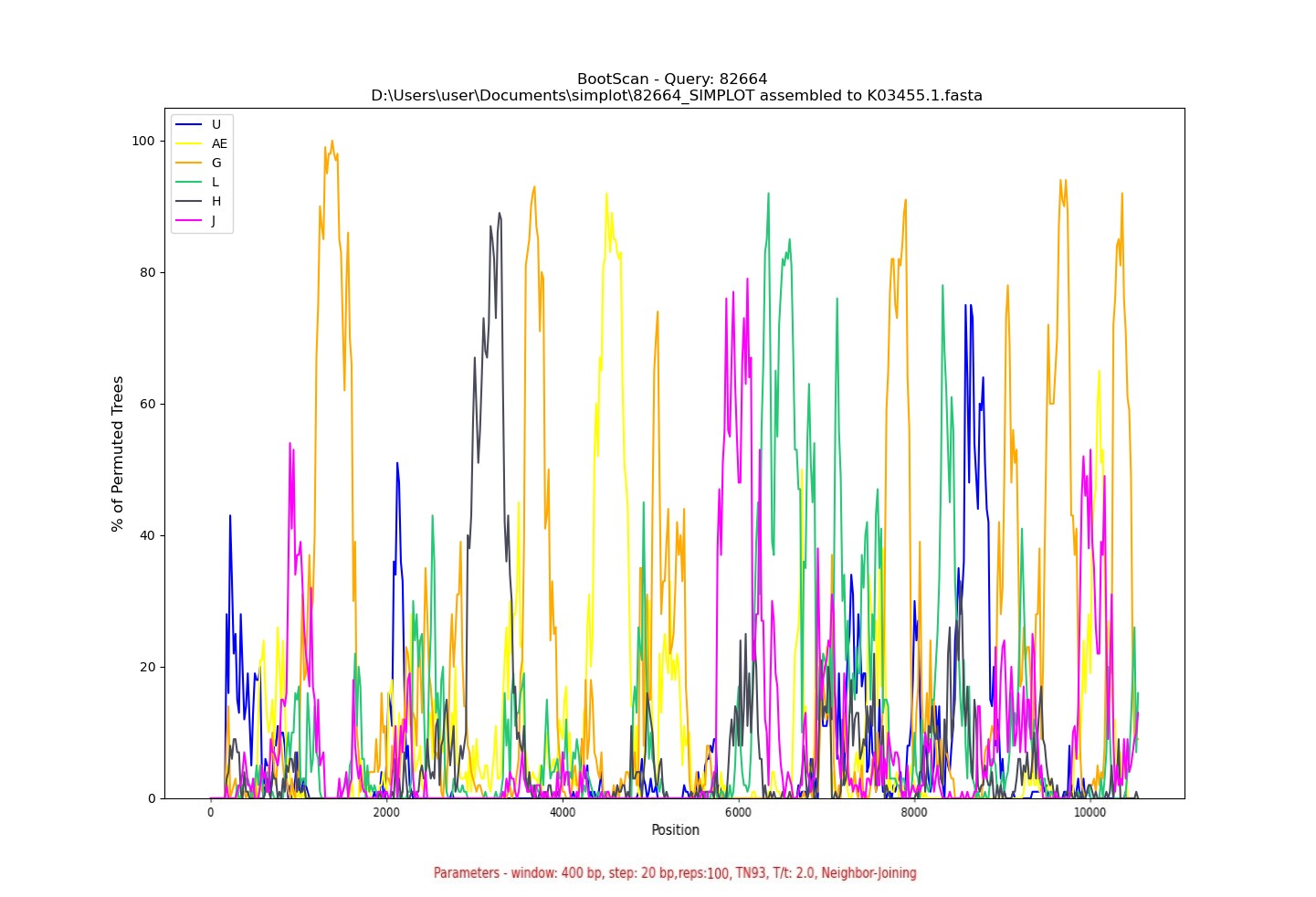


S


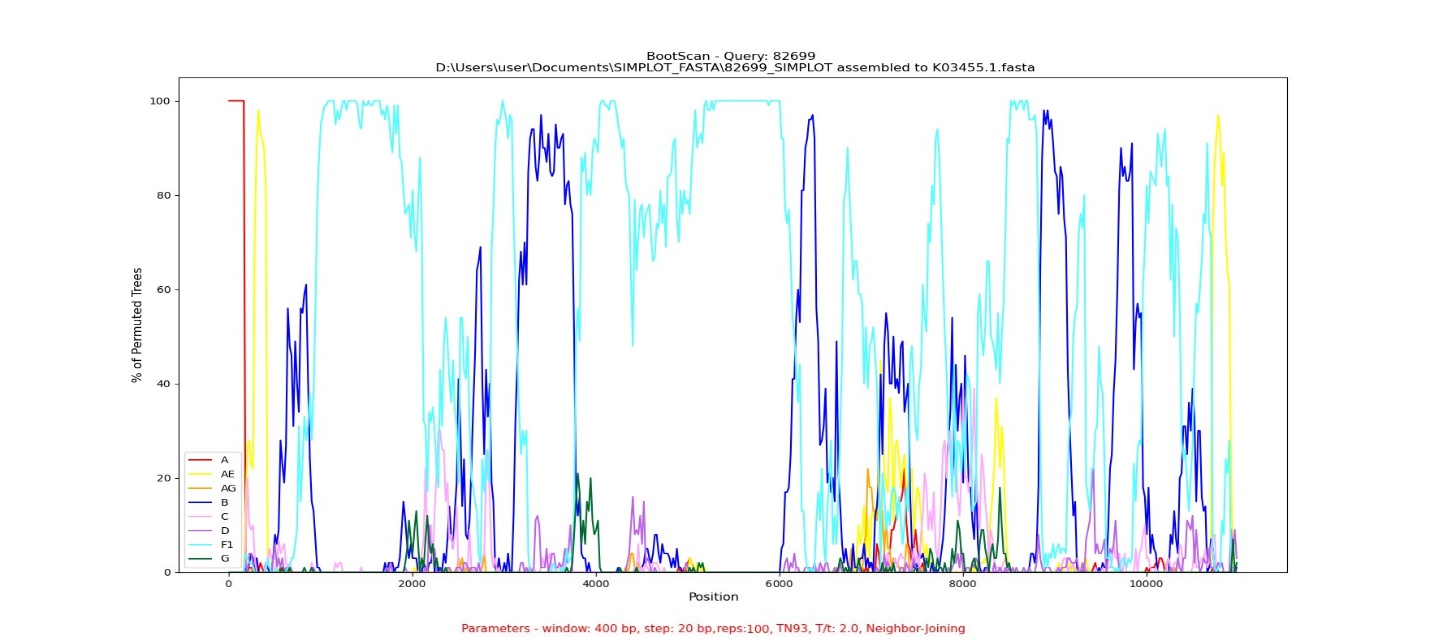


T


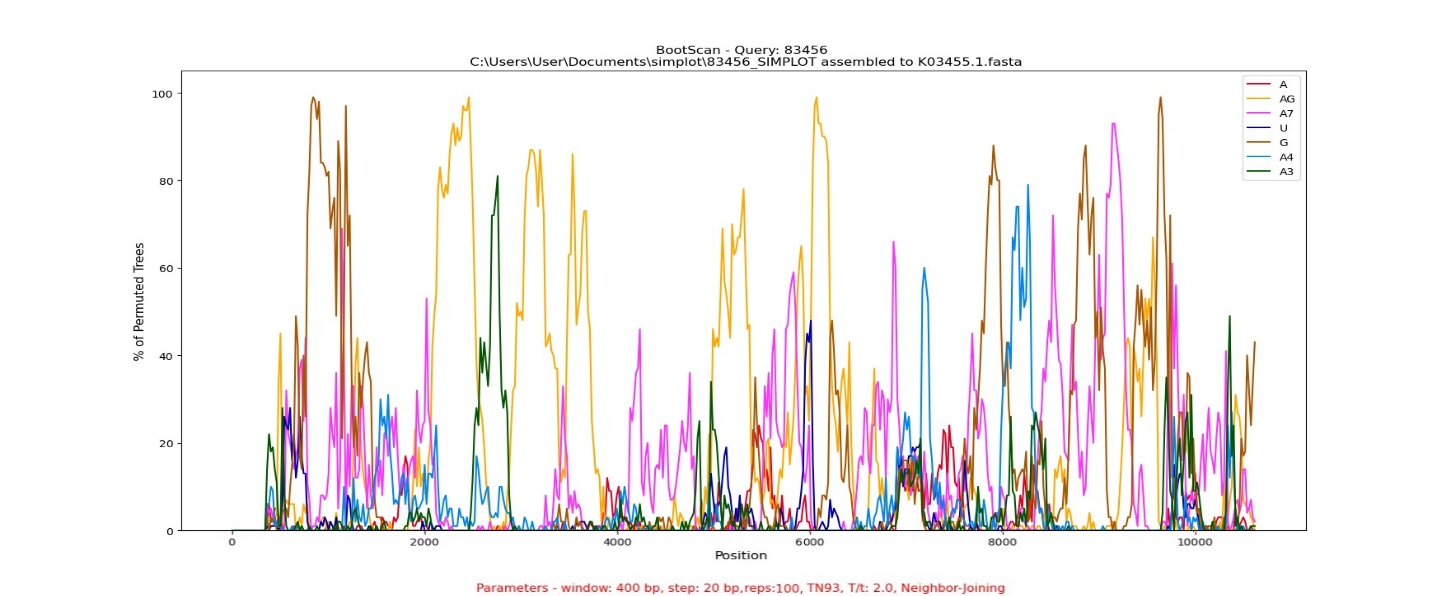


U


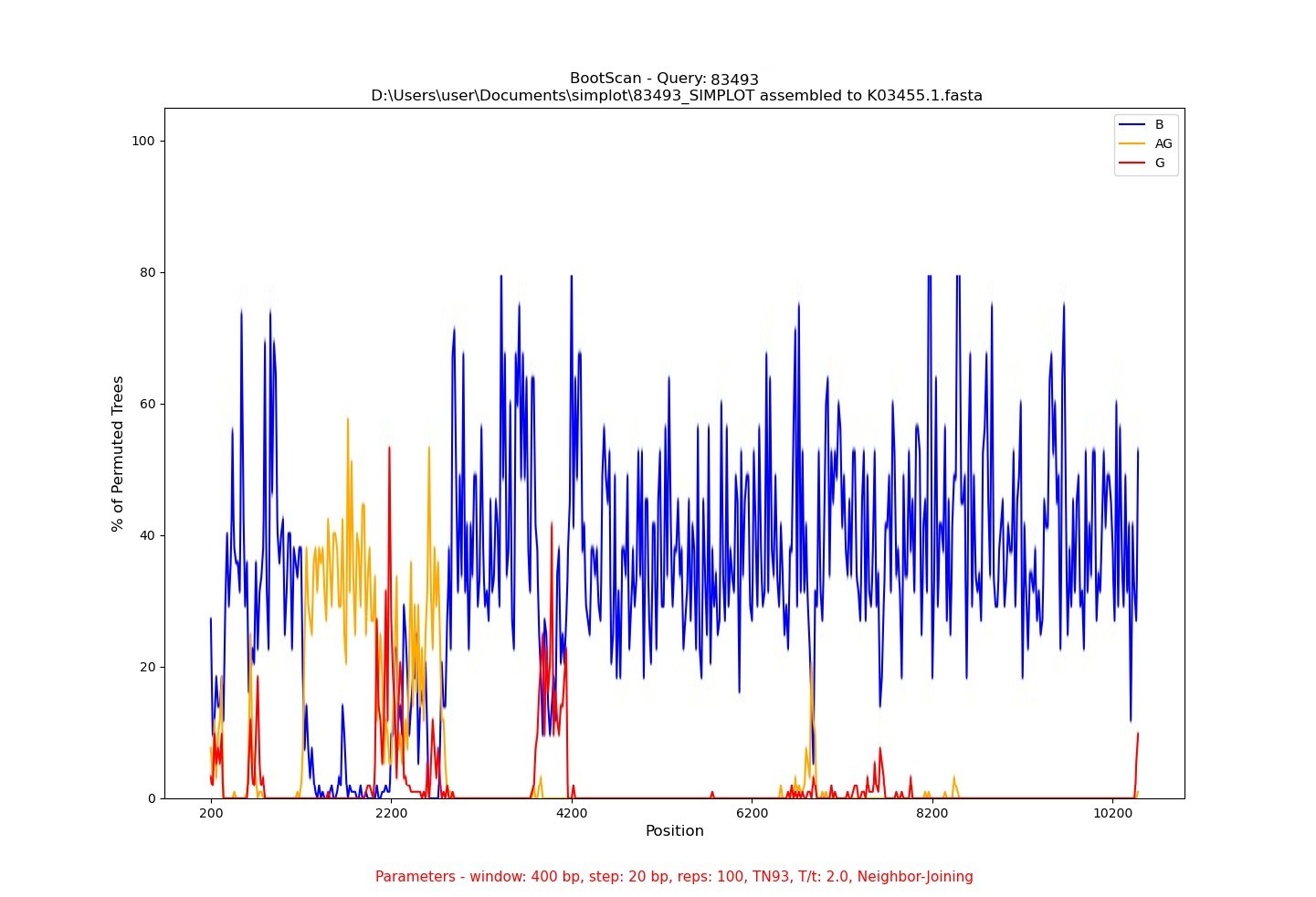


V


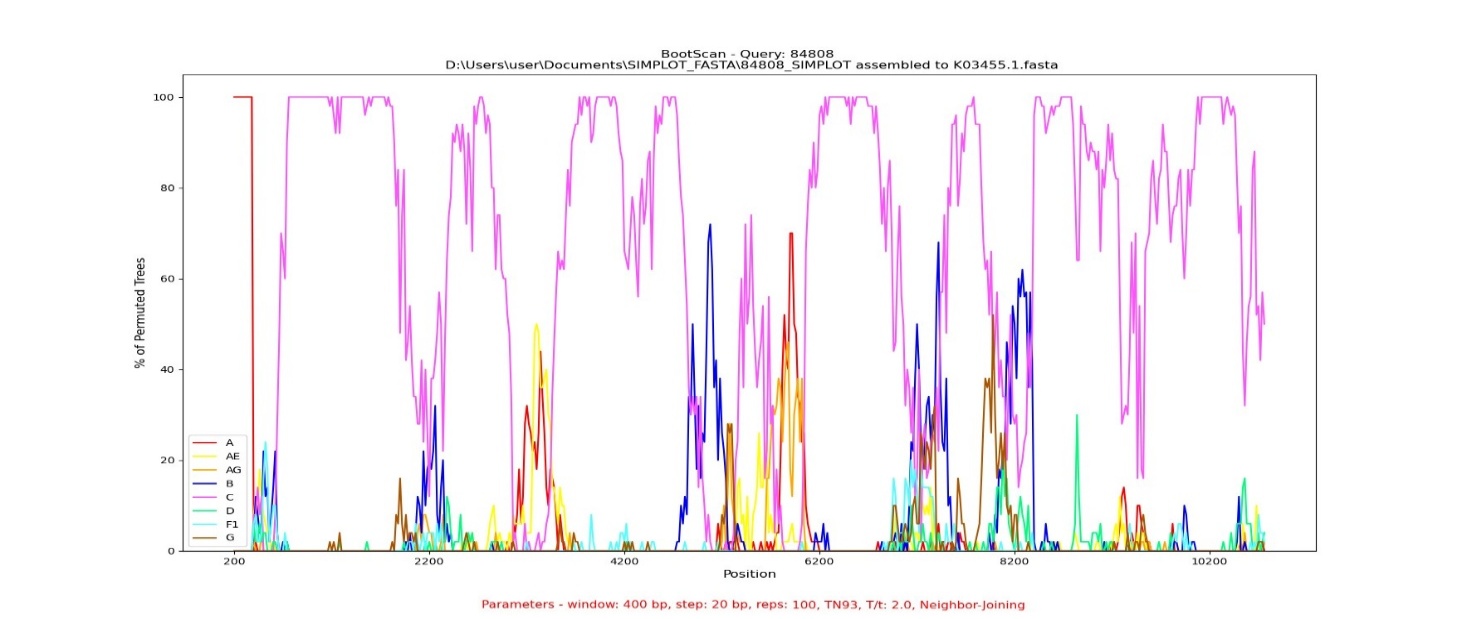


W


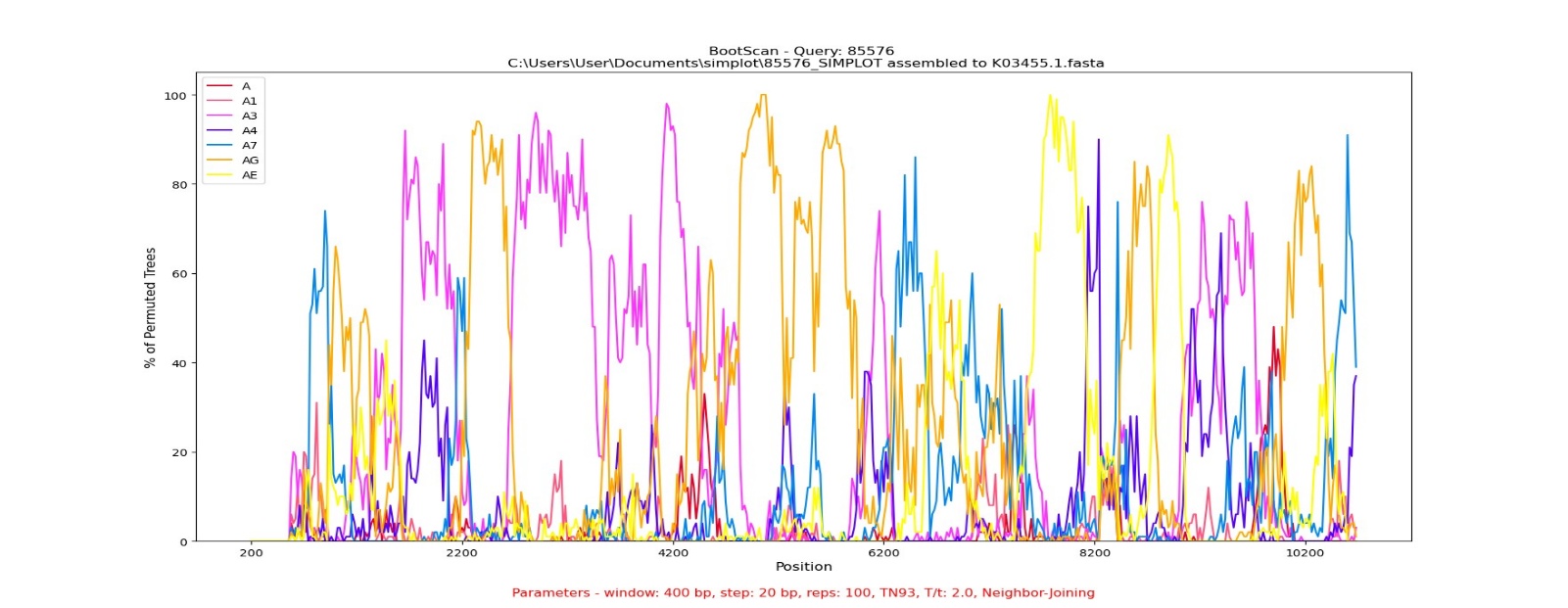


X


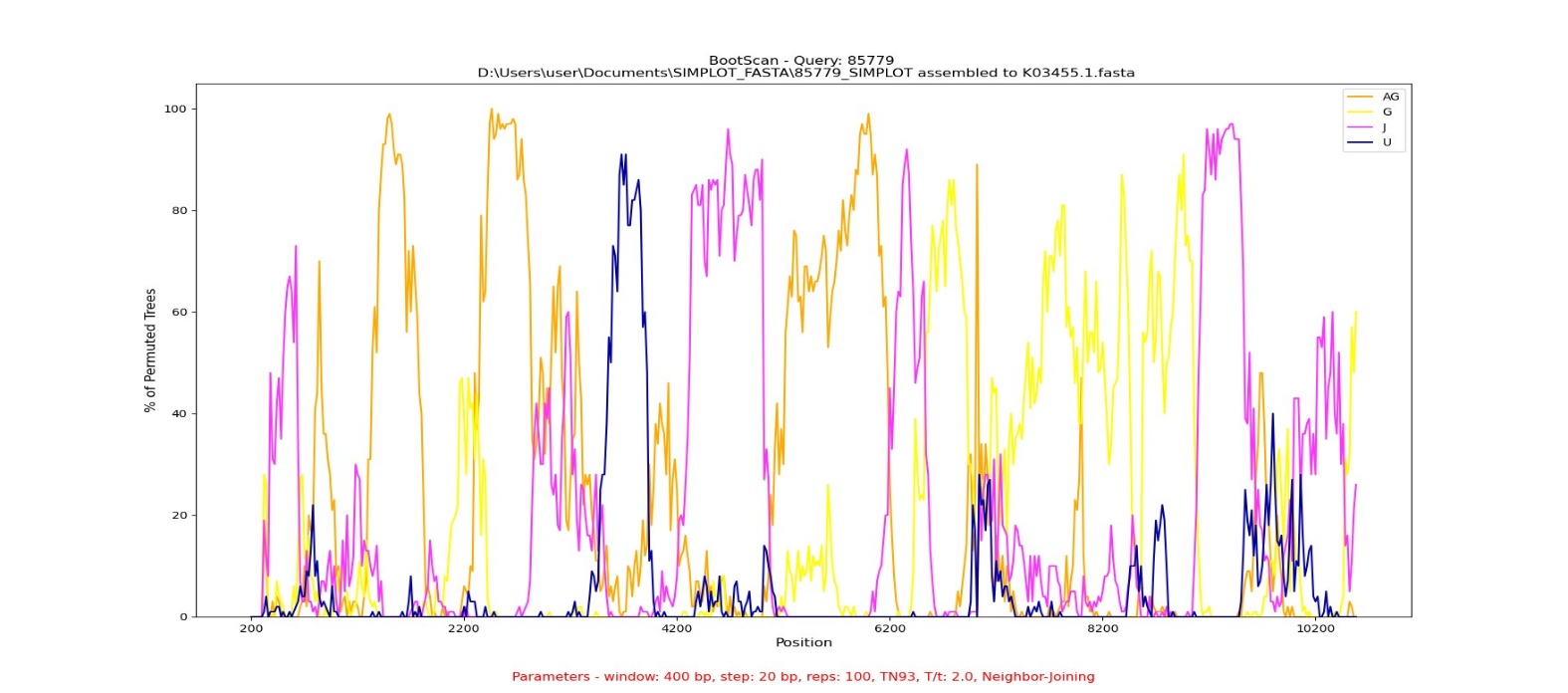


Y


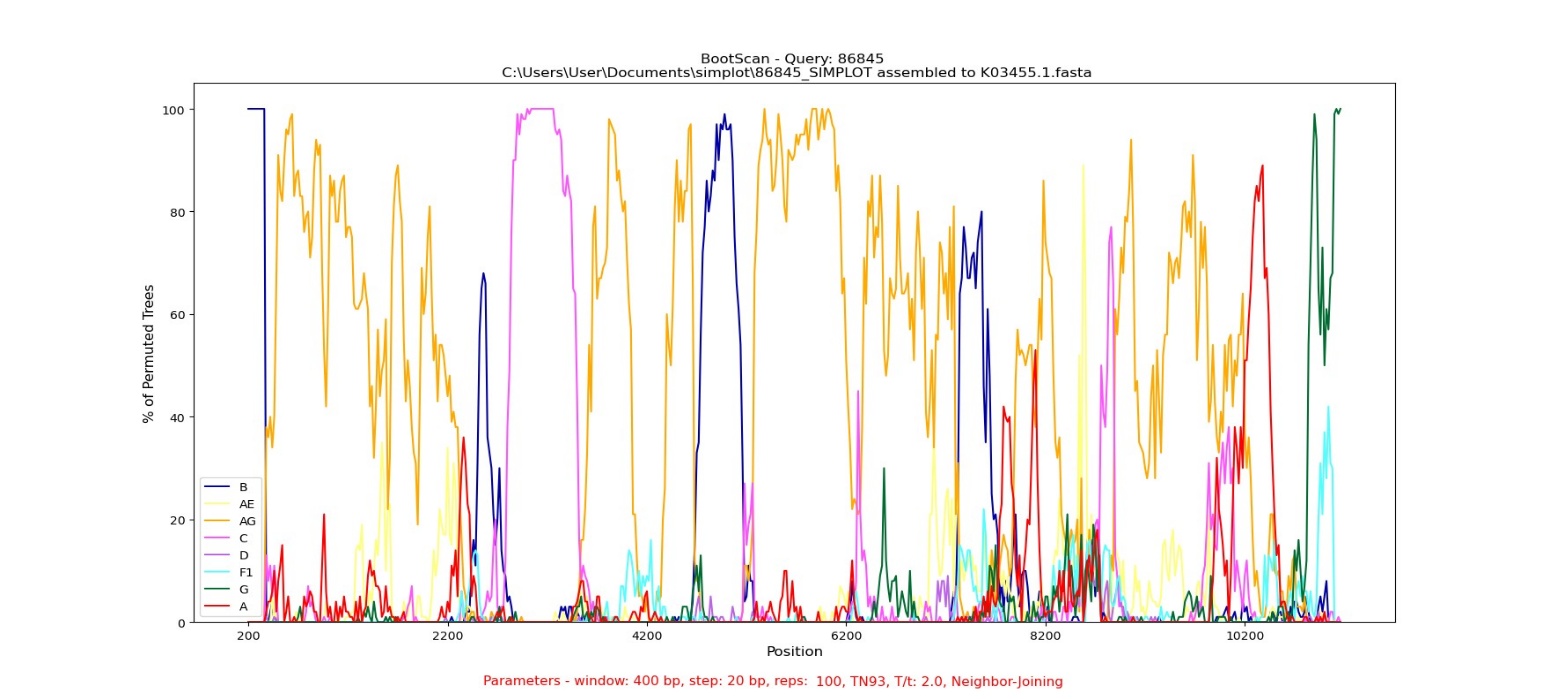


Z


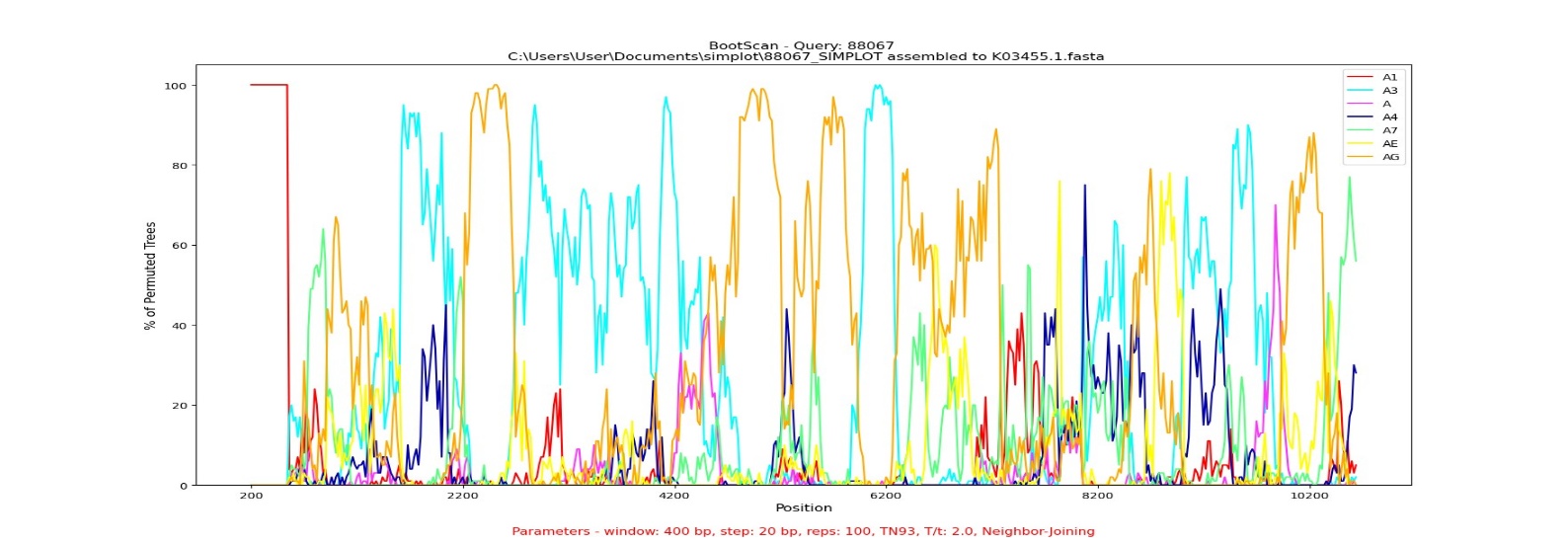


ABBB

AC


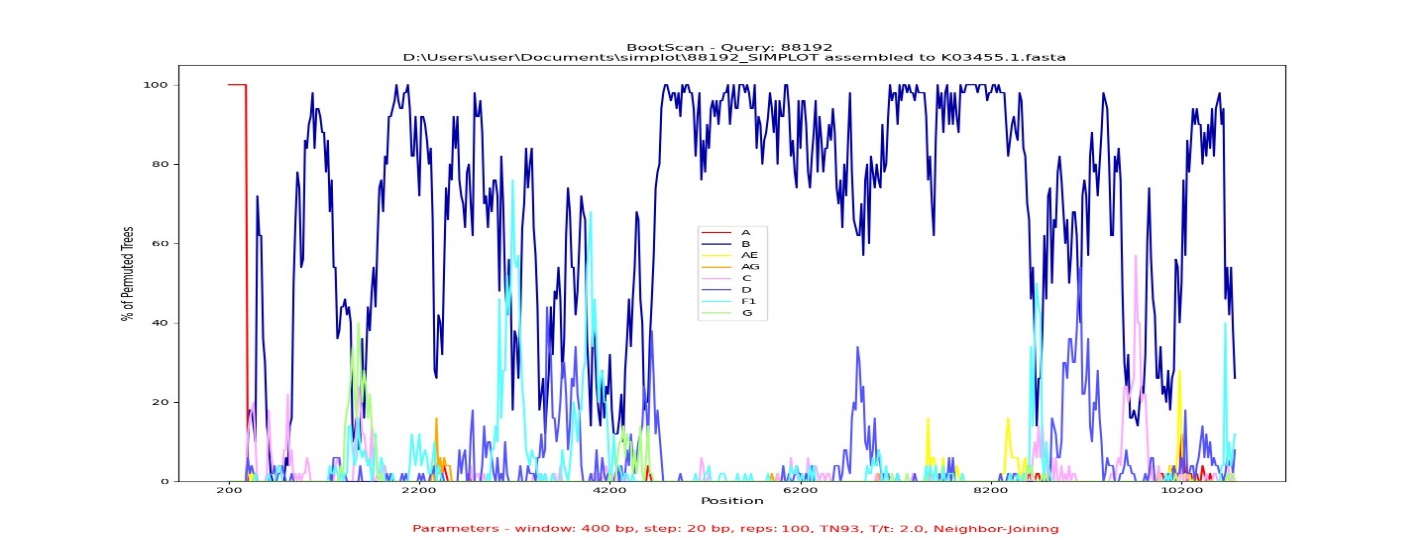


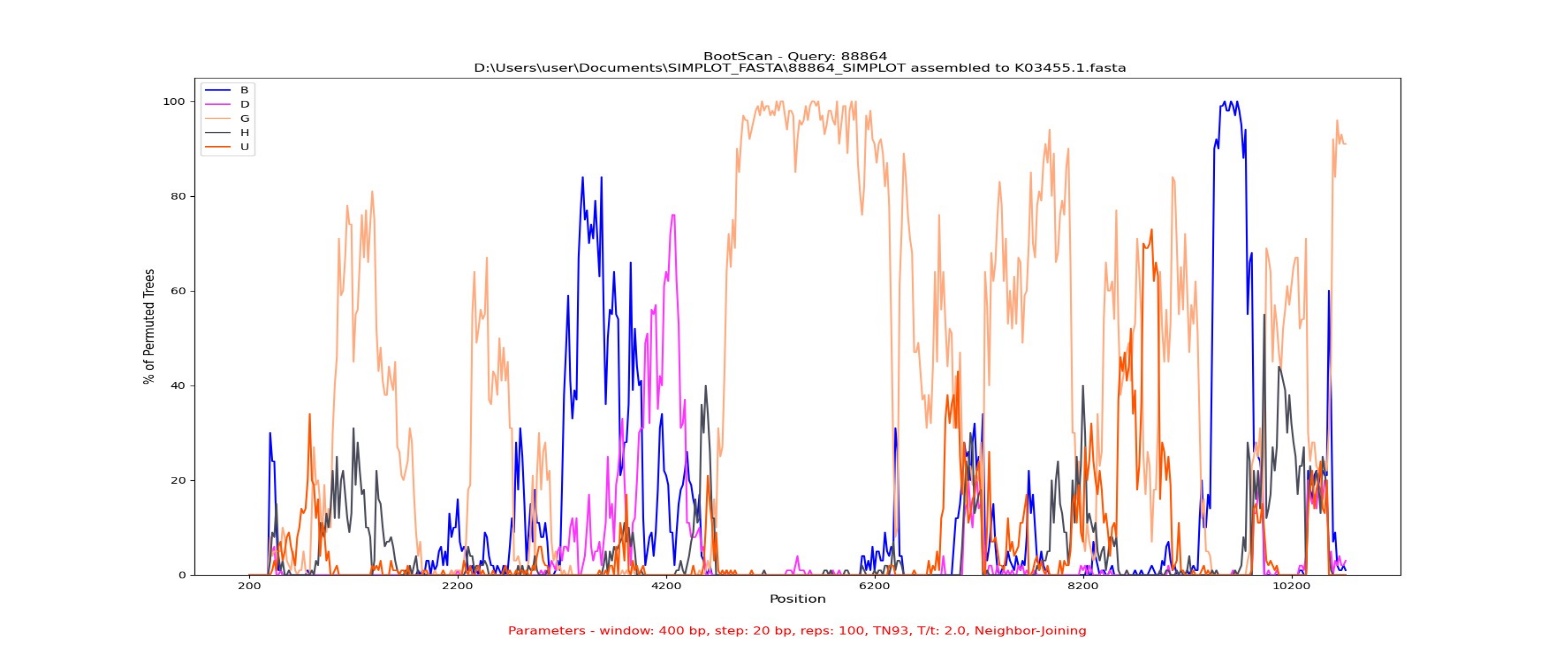


ADBB

ABBB

AC


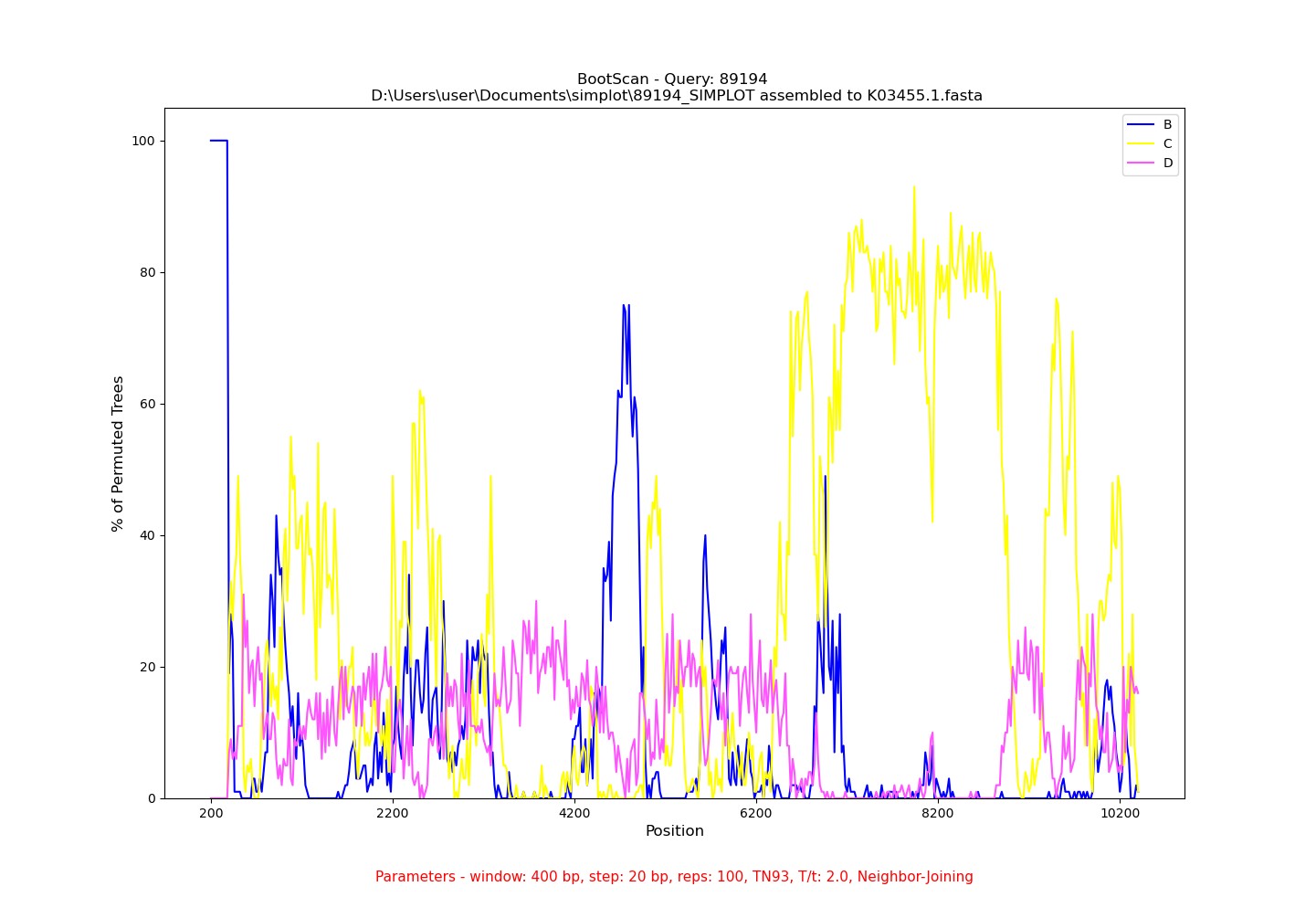


AE


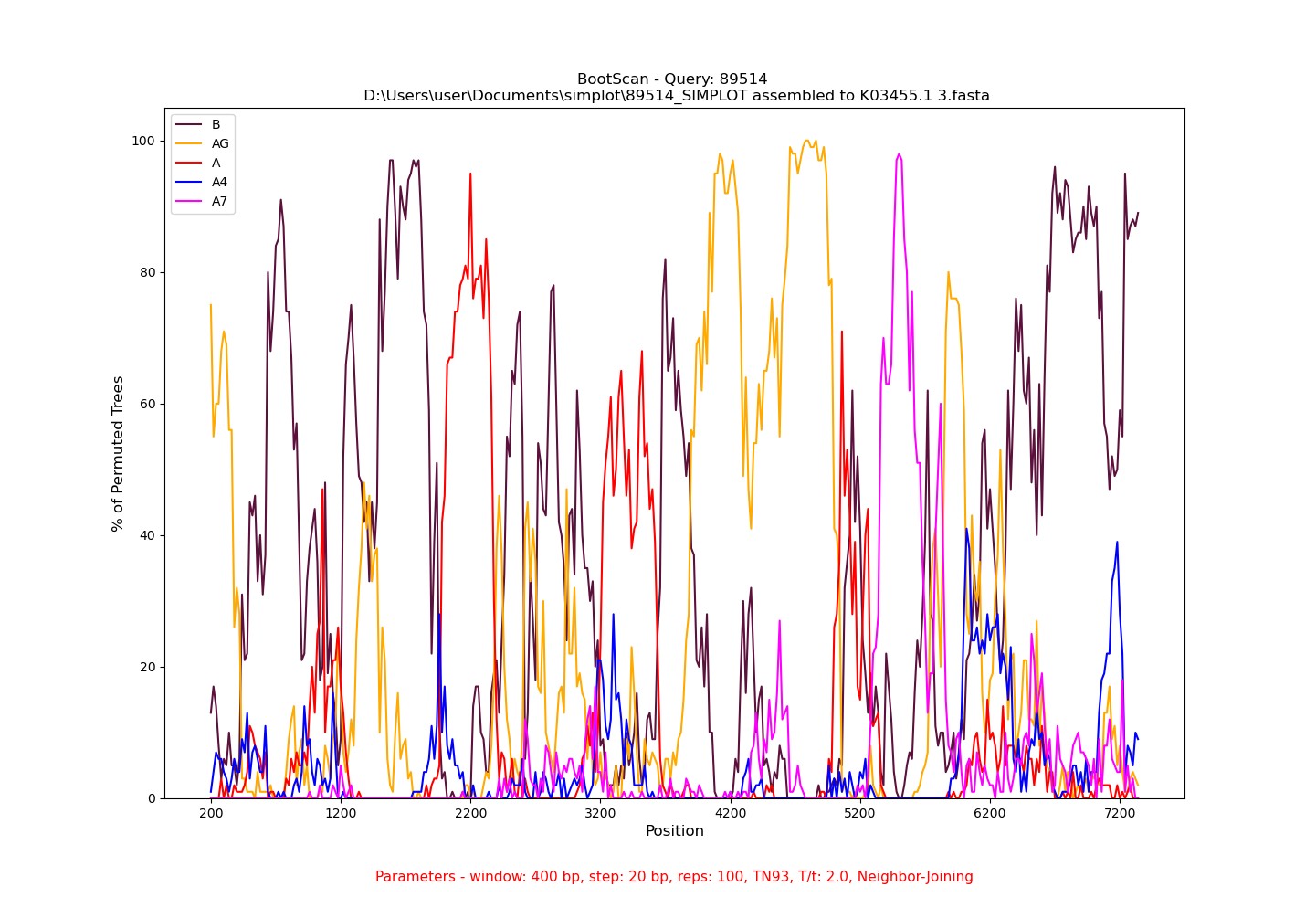


AF


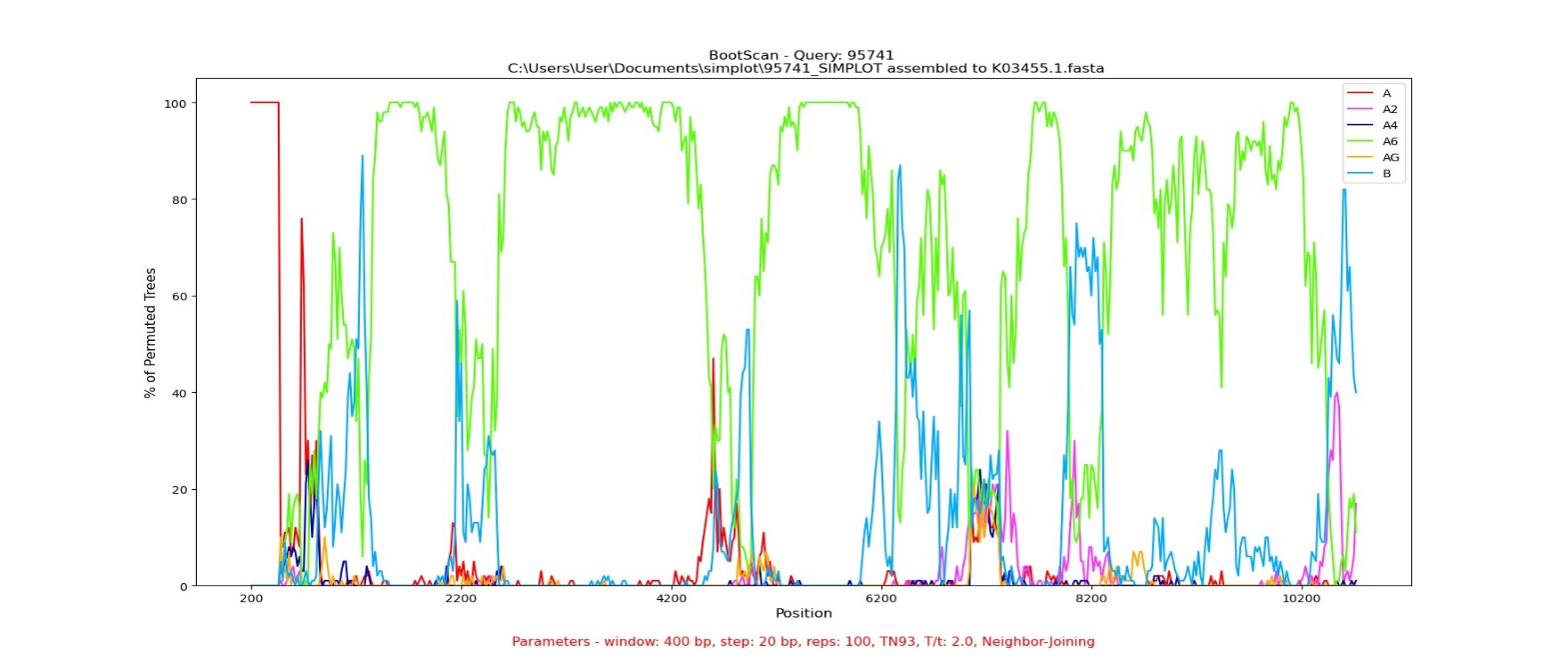


AGBB


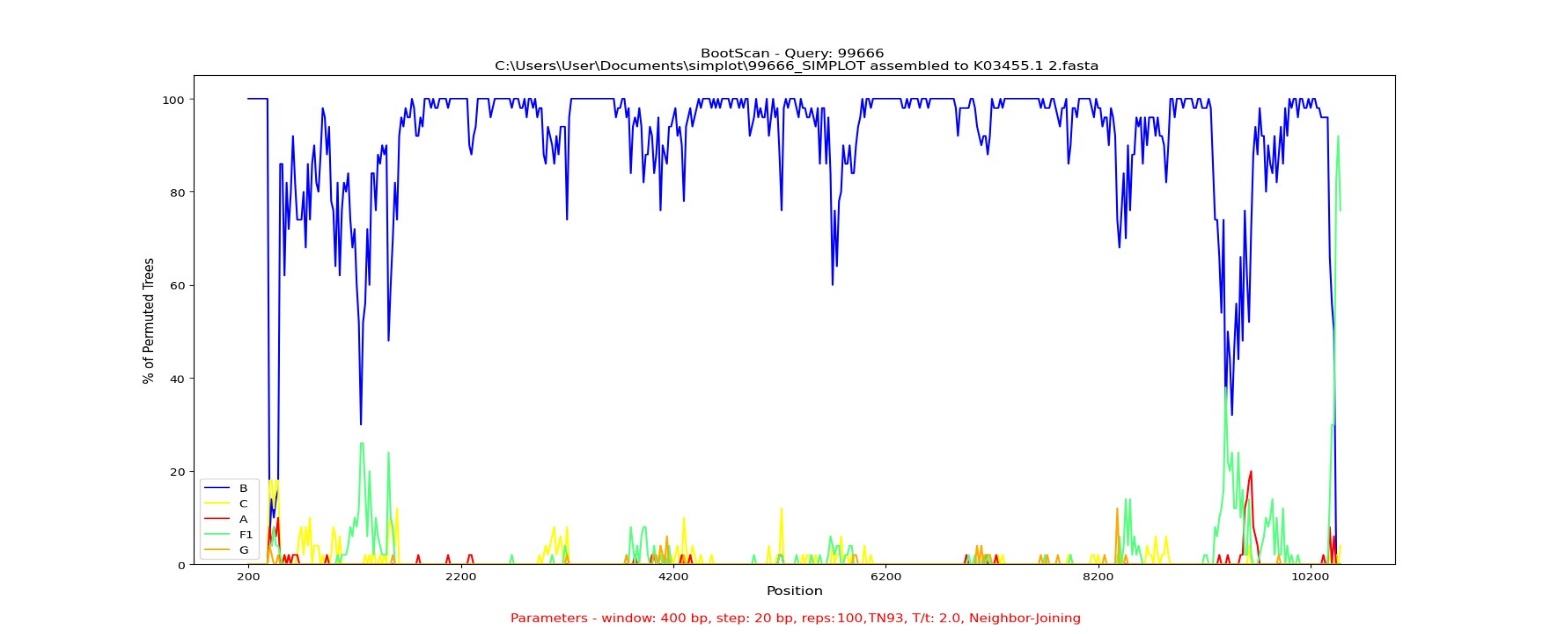

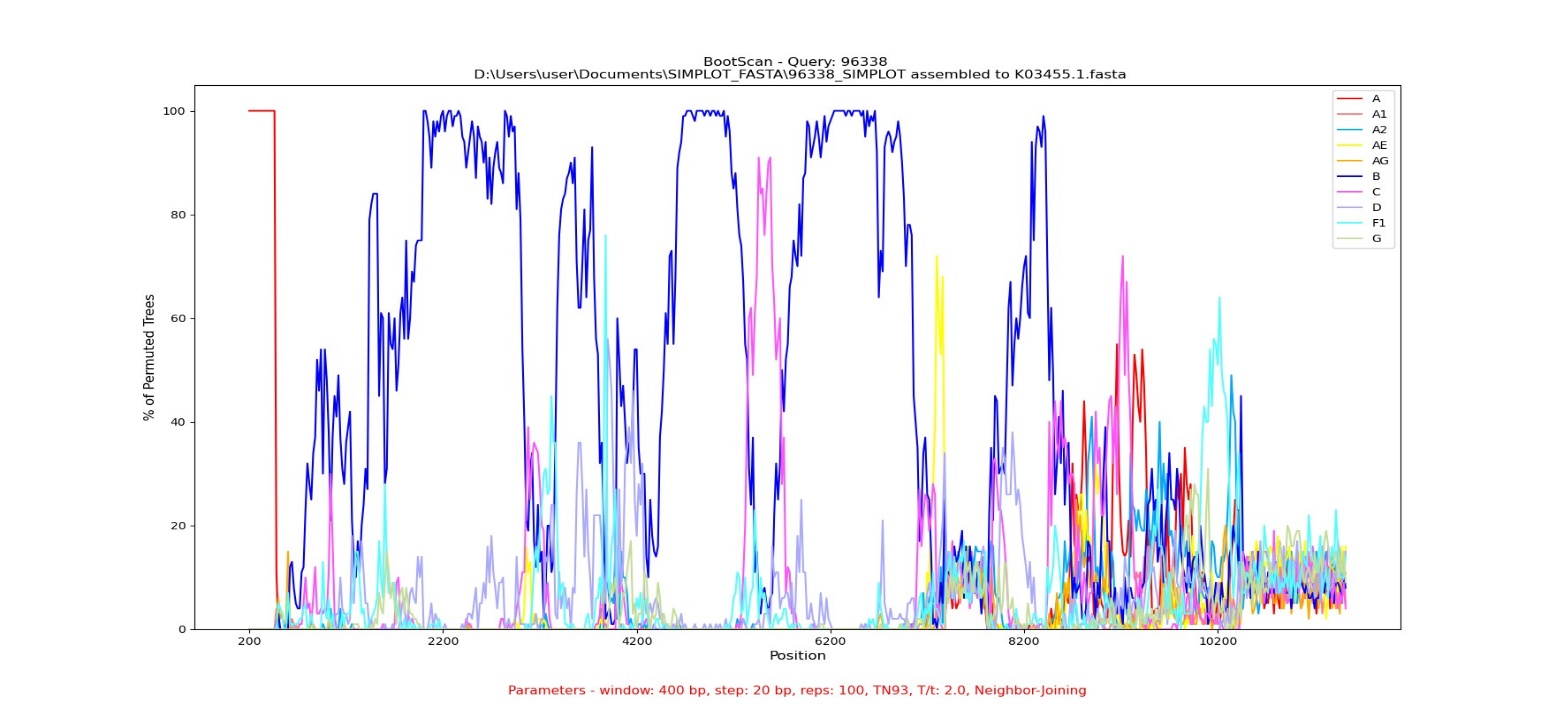


AJ

AHBB

AIBB


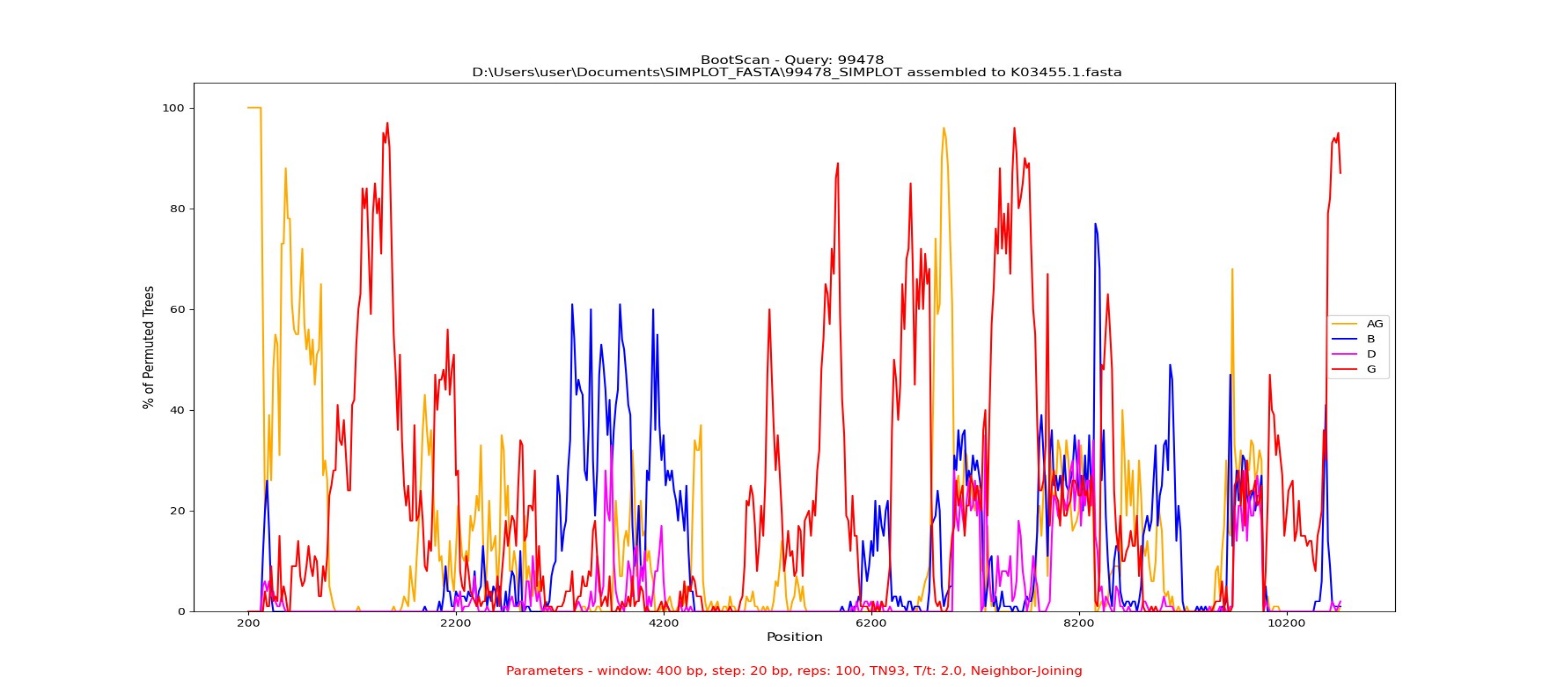


Supplement 4. Simplot++ graph output for all samples included in the Study.

HIV-1 NFLG sequences generated with the newly designed amplicons method served as input in the form of a FastA sequence to Simplot++ was used with BootScan mode and the following parameters: 100-bootstrap repetitions, at a sliding window of 400 base-pairs(bp) with a 20bp step, using the TN93 (Tamura-Nei 1993) substitution model and Neighbor-Joining (NJ) phylogeny.

The X axis is the position in the Query sequence, of note in some samples the axis starts after position 200 and ends at position 10,200, which corresponds to the ~10Kb genome size of HIV-1.

The Y axis represents percentage (out of 100%) of similarity between the query sequence to for HIV-1 subtypes reference sequences used as comparator (see legend top right corner for the color matching each viral subtype).

Each query sample was analyzed against a data set of HIV-1 pure-subtypes (A, A1, A2, A4, A6, A7, B, C, D, F1, G, H, I, J, L, U), with the exception of Recombinant Forms: AG and AE that were added as reference groups even though they are not pure subtype, due to their relatively high prevalence in the study population cohort.

Every sample was analyzed against the HIV-1 subtypes library, in each plot only prominent subtypes that showed some level of similarity to the query sequence are displayed as a colored trend line across the sequence with the corresponding viral subtype in the legend.
